# Direct Visualization of Supramolecular Binding and Separation of Light Hydrocarbons in MFM-300(In)

**DOI:** 10.1021/acs.chemmater.2c01097

**Published:** 2022-06-06

**Authors:** Lixia Guo, Mathew Savage, Joe H. Carter, Xue Han, Ivan da Silva, Pascal Manuel, Svemir Rudić, Chiu C. Tang, Sihai Yang, Martin Schröder

**Affiliations:** † Department of Chemistry, University of Manchester, Manchester M13 9PL, U.K.; ‡ Diamond Light Source, Harwell Science and Innovation Campus, Didcot OX11 0DE, U.K.; § ISIS Facility, STFC Rutherford Appleton Laboratory, Chilton OX11 0QX, Oxfordshire, U.K.

## Abstract

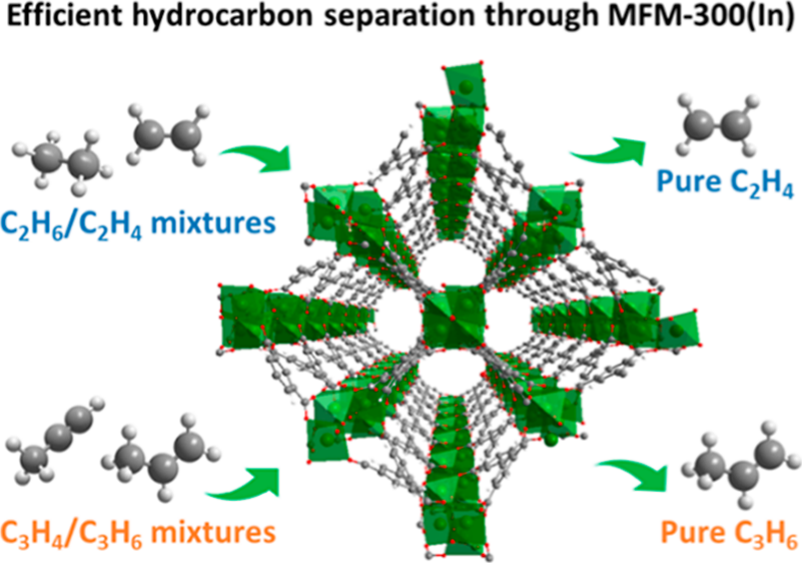

The purification of light olefins is one of the most important chemical separations globally and consumes large amounts of energy. Porous materials have the capability to improve the efficiency of this process by acting as solid, regenerable adsorbents. However, to develop translational systems, the underlying mechanisms of adsorption in porous materials must be fully understood. Herein, we report the adsorption and dynamic separation of C_2_ and C_3_ hydrocarbons in the metal–organic framework MFM-300(In), which exhibits excellent performance in the separation of mixtures of ethane/ethylene and propyne/propylene. Unusually selective adsorption of ethane over ethylene at low pressure is observed, resulting in selective retention of ethane from a mixture of ethylene/ethane, thus demonstrating its potential for a one-step purification of ethylene (purity > 99.9%). *In situ* neutron powder diffraction and inelastic neutron scattering reveal the preferred adsorption domains and host–guest binding dynamics of adsorption of C_2_ and C_3_ hydrocarbons in MFM-300(In).

## Introduction

Light olefins, primarily ethylene (C_2_H_4_) and propylene (C_3_H_6_), are the cornerstone of petrochemical industries for the production of polymers and various fine chemicals.^1^ The current global ethylene and propylene production is around 200 million tons per year.^1^ These short-chain alkenes are produced typically by the steam cracking of feedstocks derived from crude oil, such as naphtha, which is a liquid mixture of short and medium, typically comprising C_5_–C_12_ chain hydrocarbons.^2,3^ Steam cracking of naphtha produces a mixture of products, which must be separated prior to use. Most commonly, post-cracking separation is performed using cryogenic distillation operating at high pressure and low temperature (as low as −160 °C). This is thus an incredibly energy-intensive process, consuming around a third of the overall energy used in the process of ethylene production.^3^ The development of energy-efficient alternatives to cryogenic distillation can effectively reduce energy consumption as well as emissions.^4^ A possible strategy involves the use of porous materials to adsorb selectively a single component from gas mixtures (*e.g.*, alkynes and alkanes) while allowing other components to pass through. The binding of gas molecules in these materials is based often upon multiple, weak, long-range supramolecular interactions, which facilitate the removal of adsorbed species and regeneration of sorbents *via* either temperature swing or pressure swing desorption. This can operate potentially at ambient conditions and thus carries a relatively low energy penalty. Several porous adsorbents have been proposed for this application, such as ion-exchange resins,^5^ zeolites,^6,7^ and, most notably, metal–organic framework (MOF) materials.^4,8−11^

Over the past 2 decades, MOFs have been studied widely for their applications in gas separations.^12^ The ability to tune the pore size and the chemical environment of MOFs makes them excellent candidates for separating molecules with similar physical properties, such as light hydrocarbons. Several MOF materials have been proposed for application in hydrocarbon separation. These utilize different strategies including the use of open metal sites,^13−15^ specific gate opening effects,^16−18^ and kinetic size exclusion.^19−23^ Recently, an interesting computational study on the effects of pore size on the selectivity of ethane/ethylene has been reported.^24^ It was found that for a given adsorbent, separation could be controlled by altering the size of the pore along one dimension while maintaining the overall pore chemistry and structure. The purification of olefins from C_2_ or C_3_ hydrocarbon streams is considered one of the most challenging and important processes in the petrochemical industry. For the production of polymer-grade C_2_H_4_ from C_2_H_4_-selective adsorbents, an additional desorption step for the release of adsorbed C_2_H_4_ molecules is required, which adds additional energy costs *via* the application of vacuum and/or heating.^25^ In contrast, C_2_H_6_-selective adsorbents have clear advantages in the practical separation of C_2_H_6_/C_2_H_4_ owing to the direct production of polymer-grade C_2_H_4_ in one step by selective retention of C_2_H_6_.^4,26^ However, C_2_H_6_-selective adsorbents are far less common than C_2_H_4_-selective materials.^9,23,27,28^

Porous materials incorporating unsaturated metal sites (typically transition metals) can afford unique electrostatic binding sites for C_2_H_4_ or C_3_H_6_*via* π-complexation.^14,28^ Although these materials show strong host–guest interactions accompanied by a high adsorption enthalpy compared with MOFs without open metal sites,^20,29^ these materials often show limited stability, especially when exposed to humid conditions. In this context, the MFM-300 series of MOF materials^29−31^ represents a useful practical example to examine the adsorption of hydrocarbons. The MFM-300 series is a group of isostructural MOFs which are composed of biphenyl-3,3′,5,5′-tetracarboxylate (L^4–^) linkers connected to [M(μ_2_-OH)_2_]_∞_ chains in a wine-rack mode. This family of MOFs differs from other MOF materials, which have been reported for separations of light hydrocarbons, in that they utilize hydroxyl groups as the primary binding sites of adsorbed gas molecules. This may be advantageous over the reported method of relatively strong binding to open metal sites^14,32^ in that the binding energy for guest–hydroxyl interactions is significantly lower, thus making MFM-300 materials more readily regenerable and less susceptible to poisoning by moisture.

Here, we report the adsorption and breakthrough separation of C_2_ and C_3_ hydrocarbons in MFM-300(In), which exhibits an unusual selective adsorption of ethane at low pressure that is distinct from that observed for MFM-300(Al).^29^ We also describe the direct visualization of the supramolecular binding of C_2_ and C_3_ hydrocarbons within the pore by a combination of *in situ* neutron powder diffraction (NPD) and inelastic neutron scattering (INS) experiments. Breakthrough experiments confirmed the efficient separation of equimolar mixtures of C_2_H_6_/C_2_H_4_ and C_3_H_4_/C_3_H_6_ by MFM-300(In) to produce high-purity ethylene and propylene (purity > 99.9%) at room temperature.

## Experimental/Methods

### Synthesis of MFM-300(In)

H_4_L (330 mg, 1.00 mmol) and In(NO_3_)_3_·5H_2_O (585 mg, 1.50 mmol) were mixed in a dimethylformamide (DMF)/MeCN mixture (30 mL, 2:1 v/v) with conc. HNO_3_ (1.0 mL) in a 250 mL glass pressure reactor. The vessel was sealed and heated at 80 °C for 48 h. The resultant flaky white precipitate was then washed with DMF and immersed in an excess of acetone for 3 days with frequent exchange of the solvent.^33^ Yield: 347 mg (42% yield based upon solvent content from microanalysis).

### Gas-Adsorption Isotherms and Breakthrough Experiments

Gravimetric isotherms (0–1000 mbar) were recorded at 273, 283, 293, 303, and 308 K (temperature-controlled water bath) for C_2_H_2_, C_2_H_4_, C_2_H_6,_ C_3_H_4_, C_3_H_6_, and C_3_H_8_ and at 195 K (dry ice/acetone) for C_2_H_2_, C_2_H_4_, and C_2_H_6_. Data were collected using an IGA-003 system (Hiden Isochema, Warrington, UK) equipped with a turbomolecular pumping system. Acetone-exchanged samples were loaded into the system and degassed at 120 °C and 1 × 10^–6^ mbar for 20 h to give a dry, desolvated material of a typical mass of *ca.* 50 mg. Ultra-pure research-grade (99.99%) gases were purchased from Air Liquide or BOC and used as received. C_2_H_2_ was purified using dual-stage cold trap systems operated at 195 K (dry ice) and an activated carbon filter before introduction to the IGA system. Dynamic breakthrough experiments were conducted on a Hiden Isochema IGA-003 with ABR attachments and a Hiden Analytical mass spectrometer by using a fixed-bed tube packed with 750 mg of MFM-300(In) powder. The sample was heated at 120 °C under a flow of dry He for 12 h for activation and then cooled to room temperature (293 K). Single-component gas breakthrough experiments with an inlet gas flow rate of 2 mL min^–1^ diluted in a flow of He (a total flow rate of 20 mL min^–1^) were performed through a fixed-bed packed with MFM-300(In). For equimolar mixtures of hydrocarbons, the flow rate of 2.0 mL min^–1^/2.0 mL min^–1^ diluted in He (a total flow rate of 20 mL min^–1^) was applied. Dynamic breakthrough experiments for 1:99 mixtures of C_2_H_2_/C_2_H_4_, C_2_H_2_/C_2_H_6_, and C_2_H_4_/C_2_H_6_ were conducted at the rate of 0.2 mL min^–1^/19.8 mL min^–1^. All breakthrough experiments were conducted at a total flow of 20 mL min^–1^ at 293 K. The concentration of the hydrocarbon gas at the outlet was determined by mass spectrometry and compared with the inlet concentration *C*_0_, where *C*/*C*_0_ = 1 indicates complete breakthrough.

## Results and Discussion

### Material and Characterization

**Figure 1:**
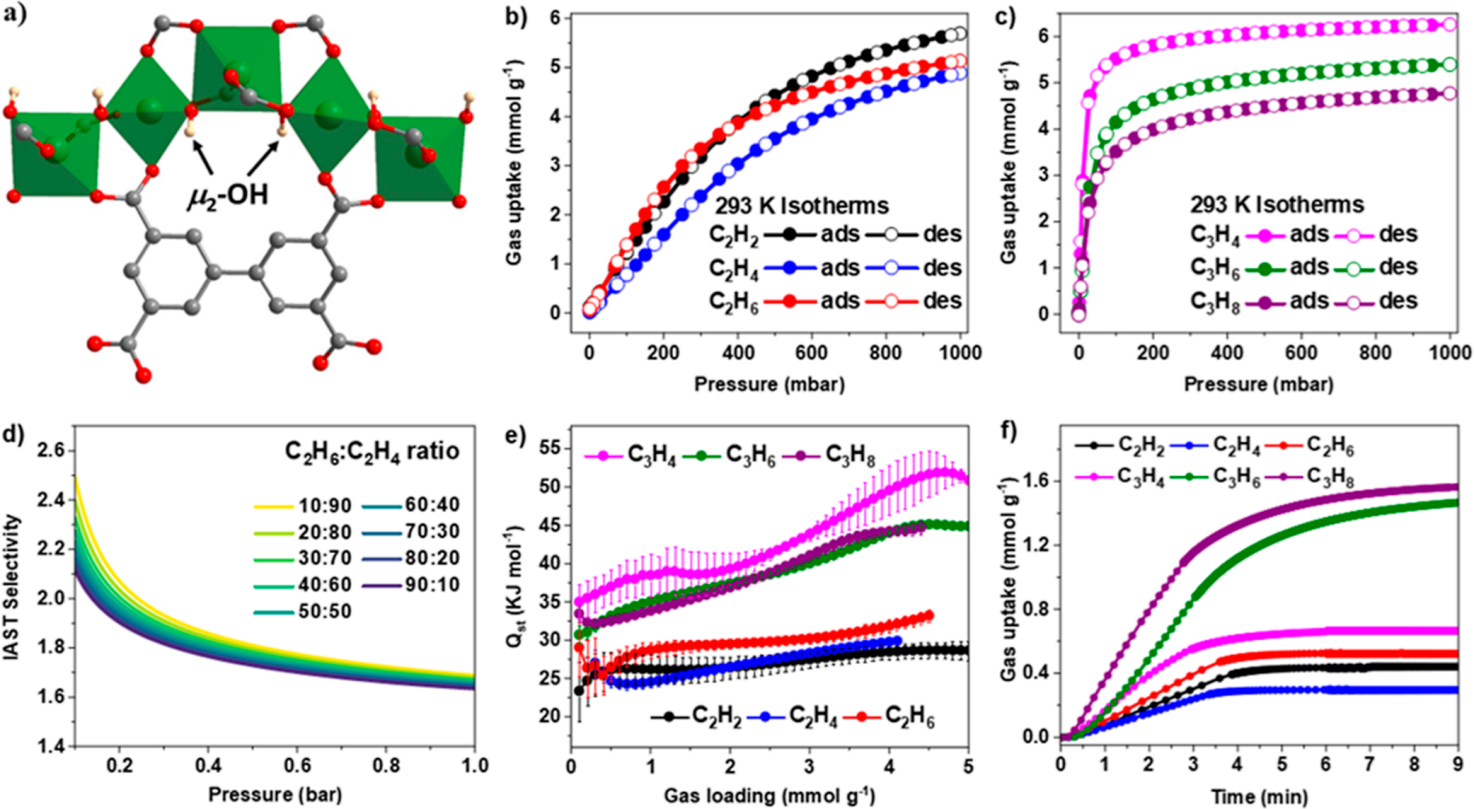
(a) View of the infinite chain of [InO_4_(OH)_2_]_∞_ linked by tetracarboxylate ligands (In: green; C: gray; O: red; H: light yellow; hydrogen atoms on the ligands are omitted for clarity). Single-component adsorption isotherms for (b) C_2_ and (c) C_3_ hydrocarbons in MFM-300(In) at 293 K. (d) Analysis of IAST selectivity of C_2_H_6_/C_2_H_4_ for MFM-300(In) at 293 K and 1 bar. (e) Isosteric heats of adsorption (*Q*_st_) for C_2_ and C_3_ hydrocarbons in MFM-300(In). (f) Adsorption kinetics of C_2_ and C_3_ hydrocarbons of MFM-300(In) at 293 K (30–70 mbar).

MFM-300(In) was synthesized by following our previously reported method^33^ (see Experimental/Methods for details). MFM-300(In) is composed of one-dimensional (1D) [In(OH)_2_O_4_]_∞_ chains bridged by tetracarboxylate ligands L^4–^ to afford a porous framework structure with channels decorated with *cis*-μ_2_-OH groups (Figure 1a). The powder X-ray diffraction pattern and thermogravimetric curves confirm the high phase purity and thermal stability of the material (Figures S1 and S2). Using N_2_ sorption isotherm data at 77 K, desolvated MFM-300(In) is found to display a Brunauer–Emmett–Teller surface area of 1030 m^2^ g^–1^ and a pore volume of 0.43 cm^3^ g^–1^ with a pore size distribution centered at 6.8 Å (Figure S3).

### Analysis of Gas-Adsorption Isotherms

C_2_ and C_3_ hydrocarbons show fully reversible uptake in MFM-300(In) with type I isotherms being observed between 195 and 303 K (Figures 1b,c, S4−S11). Single-component adsorption isotherms reveal that MFM-300(In) has a distinct binding affinity to C_3_H_4_ over C_3_H_6_ and C_3_H_8_ and to C_2_H_6_ over C_2_H_4_ over a wide range of temperatures from 273 to 303 K. The uptake of C_3_ hydrocarbons exhibits steep adsorption isotherms at low pressure, with C_3_H_4_, C_3_H_6_, and C_3_H_8_ reaching 73 to 87% of their total adsorption capacity at 1 bar at a pressure of 100 mbar; it is notable that these isotherms reach a plateau at 400 mbar at 293 K. The total adsorption capacity of these gases at 1 bar and 293 K follows the degree of unsaturation of the gas, with C_3_H_4_, C_3_H_6_, and C_3_H_8_ reaching 6.3, 5.4, and 4.8 mmol g^–1^, respectively, comparable with the highest values reported for MOF materials in the literature.^34,35^

The C_2_ hydrocarbons exhibit less steep adsorption profiles than the C_3_ analogues, reaching only 16 to 49% of their total capacity at 1 bar at 100 mbar at 293 K. Interestingly, the uptake at low pressure of the C_2_ hydrocarbons does not follow the degree of unsaturation as is observed in the isostructural MFM-300(Al).^29^ Furthermore, analysis of the isotherms by ideal adsorbed solution theory (IAST) indicates that there is a distinct reversal of the selectivities of ethane and ethylene so that MFM-300(In) exhibits selectivity toward ethane at 293 K (Figure 1d) similar to that observed in MFM-300(V^III^).^36^ This is an unusual observation considering that the In(III), V(III), and Al(III) analogues of MFM-300 have identical pore chemistry and only differ in that MFM-300(In) and MFM-300(V^III^) have a slightly larger pore diameter than MFM-300(Al).^29,33,36^ As the uptakes of ethylene in MFM-300(M) (M = In, V^III^, and Al) are similar (4.9, 6.0, and 4.3 mmol g^–1^, respectively), this phenomenon can be explained by MFM-300(In) having a greater affinity for ethane, which has a much greater uptake in the In(III) and V(III) analogues (5.1 and 7.1 mmol g^–1^) compared to Al(III) (∼0.8 mmol g^–1^) at 293 K and 1 bar. Interestingly, a recent computational study reported that a small change in the diameter of the channel was able to induce a large effect on the selectivity of ethane/ethylene.^24^ This may shed light on the observed reversal of selectivity between the ethylene-selective MFM-300(Al) and the ethane-selective MFM-300(In) as the former has a smaller pore compared to the latter (∼6.0 and 6.8 Å, respectively, determined by analysis of N_2_ isotherms at 77 K).

The isosteric enthalpy (*Q*_st_) and entropy (Δ*S*) of adsorption as a function of gas uptake were determined by fitting of the Van’t Hoff equation to the adsorption isotherms measured for each gas (Figures 1e, S12, and S13). The initial value of *Q*_st_ for C_2_H_2_ is around 25 kJ mol^–1^, and the change is relatively steady throughout the uptake process. The value of *Q*_st_ for C_2_H_6_ at near-zero coverage is 30 kJ mol^–1^, higher than that for both C_2_H_4_ and C_2_H_2_ over the entire range of loading, suggesting that MFM-300(In) exhibits a stronger binding affinity for C_2_H_6_ than C_2_H_4_ and C_2_H_2_. At the same time, *Q*_st_ for C_2_H_6_ increases continuously from 25 to 33 kJ mol^–1^ with the increase of gas loading from 0.1 to 4.0 mmol g^–1^, demonstrating the presence of strong adsorbate–adsorbate intermolecular interactions at high surface coverage, reflecting potential cooperative binding. Similar behavior has been observed in other porous sorbents.^4,37−39^ The values of *Q*_st_ for C_2_H_6_ in MFM-300(In) are significantly higher than that for MFM-300(Al), reflecting the larger pores in the former due to the larger metal center and associated lattice parameters. This allows additional C_2_H_6_ molecules to be located at optimal sites within the pore of MFM-300(In) *via* intermolecular interactions. The values of *Q*_st_ for C_2_H_6_ in MFM-300(In) are comparable with those of other reported C_2_H_6_-selective MOFs.^4,26^ The adsorption enthalpy for C_3_ hydrocarbons (30–36 kJ mol^–1^) is relatively high at low loading compared with C_2_ hydrocarbons, and C_3_H_4_ shows a higher value for *Q*_st_ compared with C_3_H_6_ and C_3_H_8_, confirming strong binding affinity of MFM-300(In) for C_3_H_4_. The adsorption kinetics for substrate uptake have been measured for MFM-300(In) (Figure 1f), and all gases exhibit rapid diffusion to reach adsorption equilibrium within 10 min. MFM-300(In) shows more rapid diffusion of C_2_H_6_ than C_2_H_2_ and C_2_H_4_, implying a kinetic selectivity for C_2_H_6_ over C_2_H_4_ and C_2_H_2_, which is beneficial for their separation under dynamic conditions. The high capacity and strong binding affinity of MFM-300(In) for C_3_H_4_ and C_2_H_6_ as well as the rapid adsorption kinetics suggest potential for the purification of mixtures of C_3_H_4_/C_3_H_6_ and C_2_H_4_/C_2_H_6_ by selective adsorption of C_3_H_4_ and C_2_H_6_, respectively.

### Breakthrough Experiments

**Figure 2:**
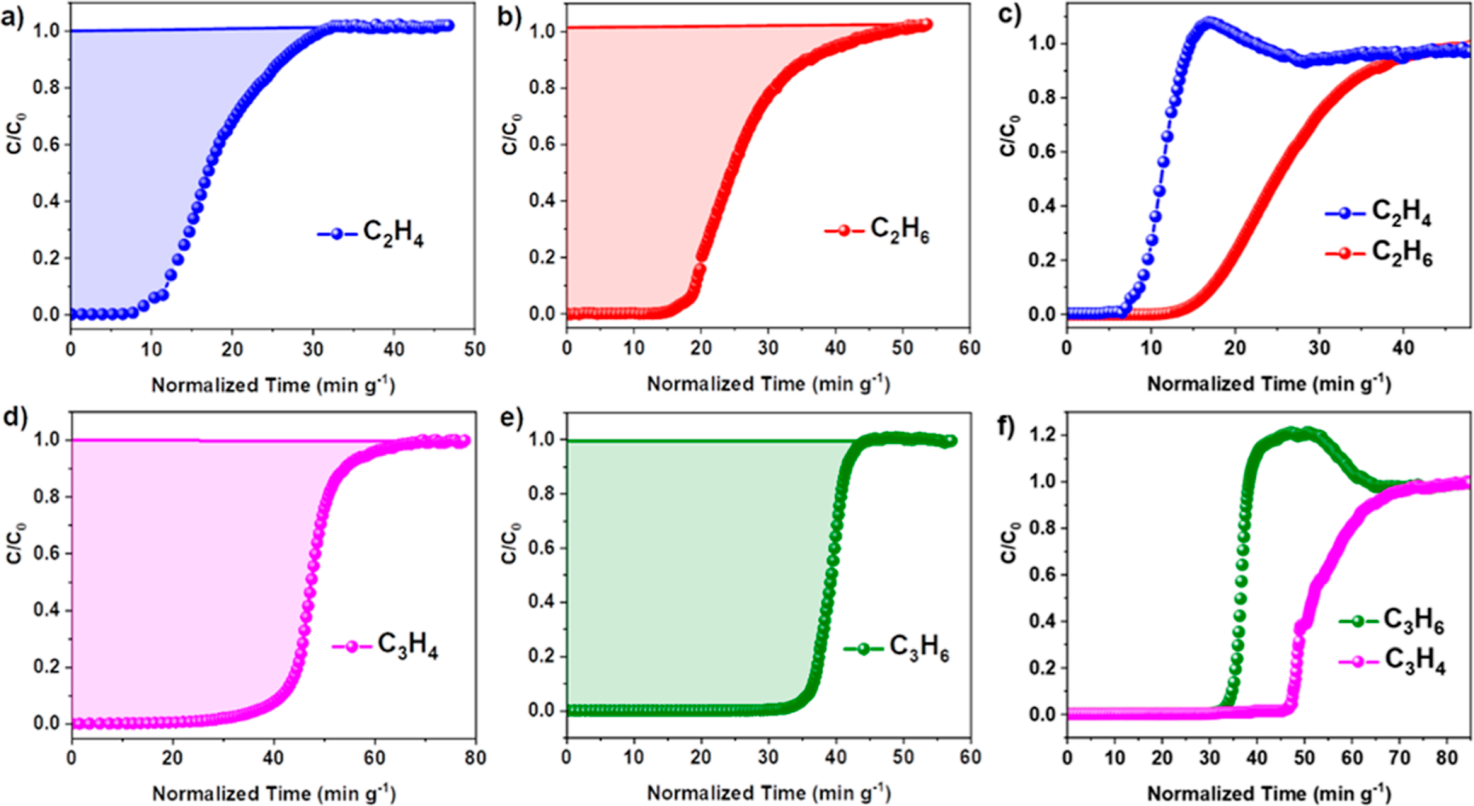
Dynamic breakthrough plots for single-component (a) C_2_H_4_, (b) C_2_H_6_, (d) C_3_H_4_, and (e) C_3_H_6_ with an inlet target gas flow rate of 2.0 mL min^–1^ diluted in He (total flow rate: 20 mL min^–1^). Dynamic breakthrough plots for equimolar mixtures of (c) C_2_H_6_/C_2_H_4_ and (f) C_3_H_4_/C_3_H_6_ with an inlet gas flow rate of 2.0 mL min^–1^/2.0 mL min^–1^ diluted in He (total flow rate: 20 mL min^–1^) through a fixed-bed packed with MFM-300(In) at 293 K.

The promising static adsorption data encouraged us to assess further the separation performance of MFM-300(In) under dynamic flow conditions. First, single-component gas breakthrough experiments were conducted to evaluate the dynamic gas adsorption (Figures 2 and S16). The dynamic adsorption capacities for each component were calculated by integrating the breakthrough curves to give dynamic uptakes of 1.4 mmol g^–1^ (C_2_H_2_), 1.0 mmol g^–1^ (C_2_H_4_), 1.6 mmol g^–1^ (C_2_H_6_), 4.4 mmol g^–1^ (C_3_H_4_), 3.5 mmol g^–1^ (C_3_H_6_), and 3.1 mmol g^–1^ (C_3_H_8_) upon saturation. These values are consistent with those obtained from static isotherm experiments.

To evaluate the feasibility of separation of C_2_ and C_3_ binary mixtures using a fixed bed packed with MFM-300(In), breakthrough experiments for equimolar mixtures of C_2_H_2_/C_2_H_4_, C_2_H_2_/C_2_H_6_, C_2_H_6_/C_2_H_4_, C_3_H_4_/C_3_H_6_, C_3_H_4_/C_3_H_8_, and C_3_H_6_/C_3_H_8_ were performed at 293 K and 1 atm. Clear separation of mixtures of C_2_H_4_/C_2_H_6_ and C_3_H_6_/C_3_H_4_ was obtained (Figure 2). In the separation of C_2_H_6_/C_2_H_4_, the breakthrough of C_2_H_4_ was observed at 6 min g^–1^, while the retention time of C_2_H_6_ was 15 min g^–1^, consistent with the analysis of unusual adsorption selectivity and high value of *Q*_st_ for C_2_H_6_. It is especially challenging to develop C_2_H_6_-selective adsorbents to enable one-step purification of C_2_H_4_ due to the common co-adsorption of C_2_H_4_ and C_2_H_6_.^27^ The profile of the breakthrough curves (Figure 2c) indicates^40^ strong competitive sorption of C_2_H_6_ over C_2_H_4_ in MFM-300(In), further confirming the high efficiency of MFM-300(In) for practical C_2_H_6_/C_2_H_4_ separation. In the case of C_3_H_4_/C_3_H_6_, the breakthrough curves indicate the sharp breakthrough of both gases with retention times of 35 and 48 min g^–1^ for C_3_H_6_ and C_3_H_4_, respectively. The apparent interval in the breakthrough time between C_3_H_4_ and C_3_H_6_ suggests that MFM-300(In) is effective for the separation of C_3_H_4_/C_3_H_6_, again consistent with the analysis of adsorption selectivity and thermodynamic data. The separation of mixtures of C_2_H_6_/C_2_H_4_ and C_3_H_4_/C_3_H_6_ by MFM-300(In) yields a productivity of 4.6 L/kg of C_2_H_4_ (purity > 99.9%) and of 16.3 L/kg of C_3_H_6_ (purity > 99.95%) at the outlet. The productivity of ethylene of MFM-300(In) is comparable with reported ethane-selective MOFs, such as IRMOF-8 (2.5 L/kg),^41^ Cu(Qc)_2_ (4.3 L/kg),^42^ and PCN-245 (5.8 L/kg)^43^ (Table S3). Thus, the efficient purification of C_2_H_6_/C_2_H_4_ and C_3_H_4_/C_3_H_6_ to produce polymer-grade olefins under the above conditions has been achieved by MFM-300(In).

### Studies of the Preferred Binding Sites and Supramolecular Interactions

**Figure 3:**
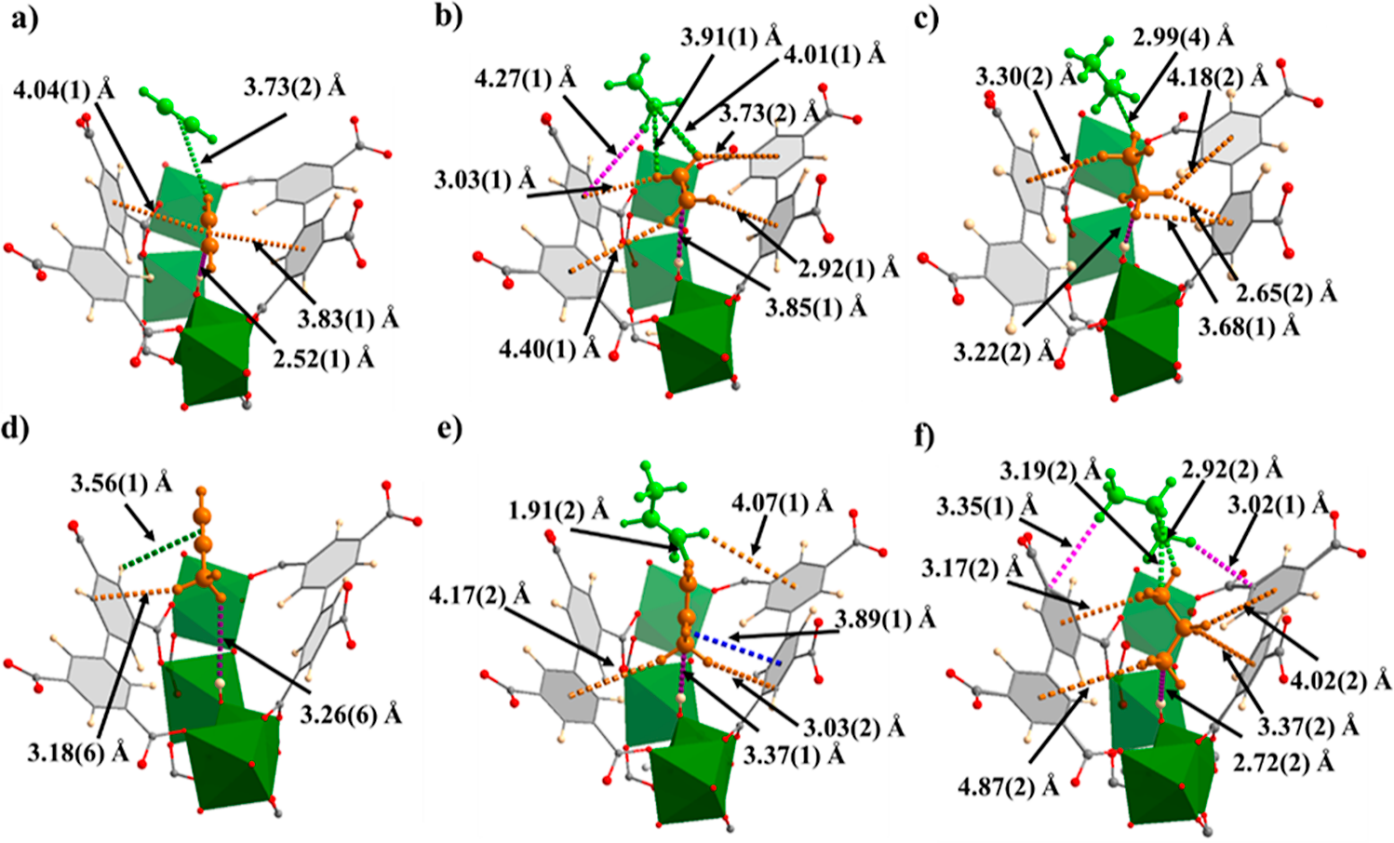
Binding sites (site I, orange; site II, green) of (a) acetylene, (b) ethylene, (c) ethane, (d) propyne, (e) propylene, and (f) propane in MFM-300(In) obtained from NPD refinements (In: green; C: gray; O: red; H: light yellow; the [InO_4_(OH)_2_] moiety is shown in green octahedron). The e.s.d. values of the bond distances are typically within 0.05 Å.

*In situ* NPD data of MFM-300(In) as a function of gas loading with C_2_D_2_, C_2_D_4_, C_2_D_6_, C_3_D_4_, C_3_D_6_, or C_3_D_8_ were refined by the Rietveld method to determine the preferred binding domains for adsorbed gas molecules within the pore. The refinements reveal two similar binding sites for all the C_2_ hydrocarbons (Figure 3), comparable to those observed in MFM-300(Al). Site I occupies a position adjacent to the bridging hydroxyl of the framework. Both unsaturated molecules show an OH···π interaction, with acetylene having a reduced H_OH_···C_2_ distance of 2.52(1) Å compared to 3.85(1) Å for ethylene, consistent with the increased polarizability of acetylene. For acetylene, this interaction is supplemented by π···π interactions between the guest molecules and the adjacent phenyl groups of the linker at distances of 3.83(1) and 4.04(1) Å. Ethylene does not have π-orbitals facing the phenyl groups of the linker and so instead exhibits electrostatic interactions between the D-centers of the ethylene and the π-system of the phenyl group at distances of between 2.92(1) and 4.40(1) Å. In contrast, ethane does not exhibit a perpendicular interaction with the bridging hydroxyl of the MOF. Instead, it displays O–H···C–D hydrogen interactions supplemented by interactions with the phenyl groups at distances between 2.65(2) and 4.18(2) Å. This mode of binding is also observed in the structure of ethane-loaded MFM-300(Al), which has a longer C_C_2_D_6__···O_OH_ distance of 3.82(1) Å compared to 3.22(2) Å in the In(III) analogue. This shorter interaction distance in MFM-300(In) suggests that the improved adsorption of ethane in MFM-300(In) is due to the presence of stronger intermolecular interactions between adsorbed ethane molecules, consistent with the greater *Q*_st_ values for ethane in MFM-300(In) over MFM-300(Al). The stronger van der Waals interactions in MFM-300(In) are likely due to the slightly larger pore size allowing the ethane molecules to orient themselves in a more favorable position compared to those in MFM-300(Al).

Binding domains of the C_3_ hydrocarbons were also determined (Figures 3d–f, S25−S27). A single binding site was found for propyne involving interactions with the hydroxyl group of the framework and the methyl group with a H_OH_–C_C_3_D_4__ distance of 3.26(6) Å. Two binding sites were found for both propylene and propane. The primary binding site displays an interaction between the methyl group of the gas molecule and the hydroxyl group of the MOF with H_OH_···C_C_3_D*x*_ distances of 3.37(1) Å for propylene and 2.72(2) Å for propane. The secondary binding sites for both propylene and propane lie more centrally in the pores of MFM-300(In). The π-orbitals of propylene site II interact with the methylene D-centers of site I *via* a T-shaped interaction at a H_C_3_H_6_(I)_–C_C_3_H_6_(II)_ distance of 1.91(2) Å. Site II of propane interacts with site I *via* van der Waals interactions between the D-centers of the respective methyl groups at distances of 2.92(2) to 3.19(2) Å.

**Figure 4:**
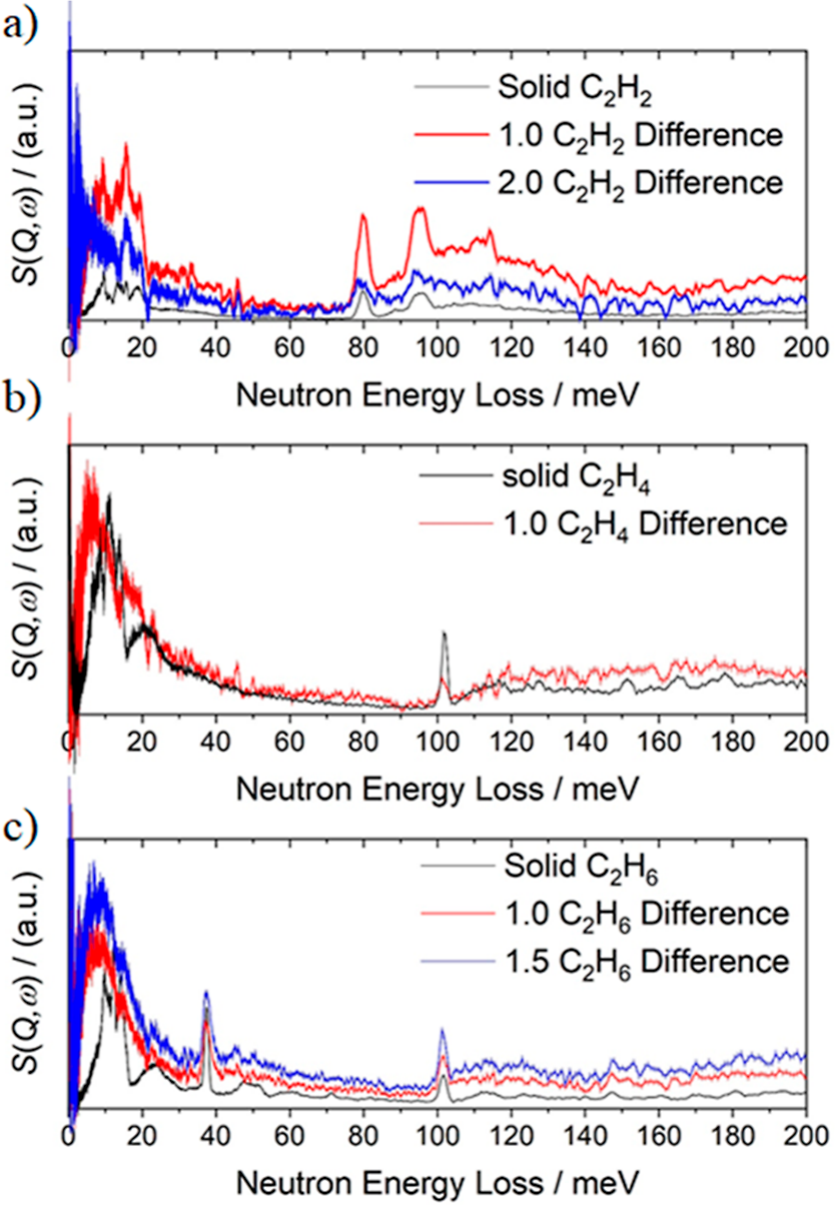
Comparison of the INS spectra of bare MFM-300(In) and MFM-300(In) loaded with (a) C_2_H_2_, (b) C_2_H_4_, and (c) C_2_H_6_. For comparison, INS spectra of the condensed gas in the solid state are also included.

The INS spectra have been obtained for MFM-300(In) as a function of gas loading (Figures 3d−f, S28−S30). For the C_2_H_2_-loaded material, the peaks in the difference spectra at around 80 and 95 meV are assigned to symmetric and asymmetric C−H bend vibrational modes of adsorbed C_2_H_2_, respectively. The peak at around 115 meV is assigned to the wagging of the C_2_H_2_ molecules, as well as to out-of-plane wagging of the four aromatic C−H groups on two benzene rings adjacent to each C_2_H_2_ molecule (Figure 4a). The spectrum of ethylene-loaded MFM-300(In) reveals a broad peak at low energy transfer (below 25 meV), which is characteristic of almost free rotational motion around the C=C axis. A peak is observed at around 100 meV, assigned to the in-plane rocking mode of −CH_2_^34^ in both the difference spectrum and that of the solid ethylene. In the spectra for ethane-loaded MFM-300(In), a broad peak is observed below 25 meV, corresponding to the almost free rotational mode around the C–C axis. Two peaks are also observed in the difference spectra which align with peaks in the spectrum of solid ethane. A peak at 37 meV is assigned to the −CH_3_ torsion, whereas the peak at 101 meV can be assigned to the CH_3_ rocking motion.^33^ The relatively high intensity of the −CH_3_ rotational mode compared to that observed in MFM-300(Al) indicates that the larger pore of MFM-300(In) and the shorter H_OH_···C_2_D_6_ distance provide a greater degree of free rotation of the ethane molecule at site I, thus decreasing the entropic penalty for adsorption and driving further uptake of ethane in MFM-300(In) compared to MFM-300(Al).

## Conclusions

An understanding of the detailed effect that pore size has on the selectivity of ethane/ethylene is important to the design of improved materials for the separation of light hydrocarbons. The MFM-300 materials provide a useful platform to investigate changes in pore size while maintaining identical pore chemistry. This study has shown that the MFM-300(In) with slightly larger pores exhibits the opposite IAST selectivity of ethane/ethylene to MFM-300(Al). Analysis of *Q*_st_ of these two gases along with NPD studies has revealed that the reason for the reversal is that the slightly larger pores of MFM-300(In) allow ethane to sit in a more favorable position, which allows for a greater degree of host–guest and guest–guest interactions, thus increasing the overall uptake. MFM-300(In) exhibits excellent separation of mixtures of C_2_H_6_/C_2_H_4_ and C_3_H_4_/C_3_H_6_ as demonstrated by dynamic breakthrough experiments, allowing the production of polymer-grade C_2_H_4_ and C_3_H_6_ (purity > 99.9%) in a one-step approach. The understanding of structure−property relationships will inform the design of future efficient sorbent materials for important separations of gas mixtures.

## Supporting Information Available

Synthesis procedures, characterization, and additional analysis of crystal structures and adsorption results (PDF)
Crystal structure data for MFM-300(In)·C_2_D_2_ (CIF)
Crystal structure data for MFM-300(In)·C_2_D_4_ (CIF)
Crystal structure data for MFM-300(In)·C_2_D_6_ (CIF)
Crystal structure data for MFM-300(In)·C_3_D_4_ (CIF)
Crystal structure data for MFM-300(In)·C_3_D_6_ (CIF)
Crystal structure data for MFM-300(In)·C_3_D_8_ (CIF)

## Supplementary Material

cm2c01097_si_001.pdf

cm2c01097_si_002.cif

cm2c01097_si_003.cif

cm2c01097_si_004.cif

cm2c01097_si_005.cif

cm2c01097_si_006.cif

cm2c01097_si_007.cif
