# Supplementary material for: Direct Visualization of Supramolecular Binding and Separation of Light Hydrocarbons in MFM-300(In)
Source: Chem Mater. 2022 Jun 6;34(12):5698–705. doi: 10.1021/acs.chemmater.2c01097 (PMC9245183; doi:10.1021/acs.chemmater.2c01097)
Supplement: Supplementary file 1 — cm2c01097_si_001.pdf [file cm2c01097_si_001.pdf]

Supporting Information

Direct visualisation of supramolecular binding and separation of light hydrocarbons in MFM-300(In)

Lixia Guo,[1§] Mathew Savage,[1§] Joe H. Carter,[1,2§] Xue Han,[1] Ivan da Silva,[3] Pascal Manuel,[3] Svemir Rudić[3], Chiu C. Tang,[2] Sihai Yang[1]* and Martin Schröder[1]*

[1] Department of Chemistry, University of Manchester, Manchester, M13 9PL (UK)

[2] Diamond Light Source, Harwell Science and Innovation Campus, Didcot OX11 0DE (UK)

[3] ISIS Facility, STFC Rutherford Appleton Laboratory, Chilton, Oxfordshire, OX11 0QX (UK)

Contents of Supplementary Information

1. Experimental

2. Powder X-Ray Diffraction

3. Thermo Gravimetric Analysis

4. Characterisation of Porosity

5. Additional Gas Adsorption Isotherms

6. Comparison of CH_4, C_2H_2, C_2H_4, C_2H_6, C_3H_4, C_3H_6 and C_3H_8 isotherms

7. Analysis and Derivation of the Isosteric Heats of Adsorption

8. Calculation of IAST Selectivity for Gas Adsorption

9. Breakthrough Experiments

10. Comparsion of Separation Performance of State-of-the-art MOFs

11. Neutron Powder Diffraction

12. Inelastic Neutron Scattering Measurement

13. References

1. Experimental

Synthesis of MFM-300(In). H_4L (330 mg, 1.00 mmol), $In(NO_3)_3 \cdot 5H_2O$ (585 mg, 1.50 mmol) were mixed in a DMF/MeCN mixture (30 ml, 2:1 v/v) with conc. HNO_3 (1.0 mL) in a 250 mL glass pressure reactor. Then the vessel sealed and heated at 80 °C for 48 h. The resultant flaky white precipitate was then washed with DMF and immersed in an excess of acetone for 3 days with frequent exchange of solvent.[1] Yield: 347 mg (42% yield based upon solvent content from microanalysis).

Gas Adsorption Isotherms and Breakthrough Experiments. Gravimetric isotherms (0-1000 mbar) were recorded at 273, 283, 293, 303, and 308 K (temperature controlled water-bath) for C_2H_2, C_2H_4, C_2H_6, C_3H_4, C_3H_6 and C_3H_8 and at 195 K (dry ice/acetone) for C_2H_2, C_2H_4, C_2H_6. Data were collected using an IGA-003 system (Hiden Isochema, Warrington, UK) equipped with a turbomolecular pumping system. Acetone exchanged samples were loaded into the system and degassed at 120 °C and 1×10^{-6} mbar for 20 h to give a dry, desolvated material of typical mass ca. 50 mg. Ultra-pure research grade (99.99 %) gases were purchased from Air Liquide or BOC and used as received. C_2H_2 was purified by dual-stage cold trap systems operated at 195 K (dry ice) and an activated carbon filter before introduction to the IGA system. Dynamic breakthrough experiments were conducted on a Hiden Isochema IGA-003 with ABR attachments and a Hiden Analytical mass spectrometer by using a fixed-bed tube packed with 750 mg of MFM-300(In) powder. The sample was heated at 120 ºC under a flow of dry He for 12h for activation, and then cooled to room temperature (293 K). Single-component gas breakthrough experiments with an inlet gas flow rate of 2 mL min^{-1} diluted in a flow of He (total flow rate of 20 mL min^{-1}) were measured through a fixed-bed packed with MFM-300(In). For equimolar mixtures of hydrocarbons, the flow rate of 2.0 mL min^{-1}/2.0 mL min^{-1} diluted in He (total flow rate of 20 mL min^{-1}) was applied. Dynamic breakthrough experiments for 1:99 mixtures of C_2H_2/C_2H_4, C_2H_2/C_2H_6, and C_2H_4/C_2H_6 were conducted at the rate of 0.2 mL min^{-1}/19.8 mL min^{-1}. All breakthrough experiments were conducted at a total flow of 20 mL min^{-1} at 293 K. The concentration of hydrocarbon gas at the outlet was determined by mass spectrometry and compared with the inlet concentration C_0, where C/C_0 = 1 indicates complete breakthrough.

Table S1. Physical parameters for C_2 and C_3 hydrocarbons.[2-8]

Gas	Molecular size (Å^3)	Boiling point (K)	Kinetic diameter (Å)
C_2H_2	3.3 × 3.3 × 5.7	188.40	3.3
C_2H_4	3.3 × 4.2 × 4.8	169.42	4.2
C_2H_6	3.8 × 4.1 × 4.8	184.55	4.4
C_3H_4	4.0 × 4.1 × 6.5	249.8	4.8
C_3H_6	4.2 × 5.3 × 6.4	225.46	4.7
C_3H_8	4.2 × 4.8 × 6.8	231.02	4.3-5.1

2. Powder X-ray Diffraction

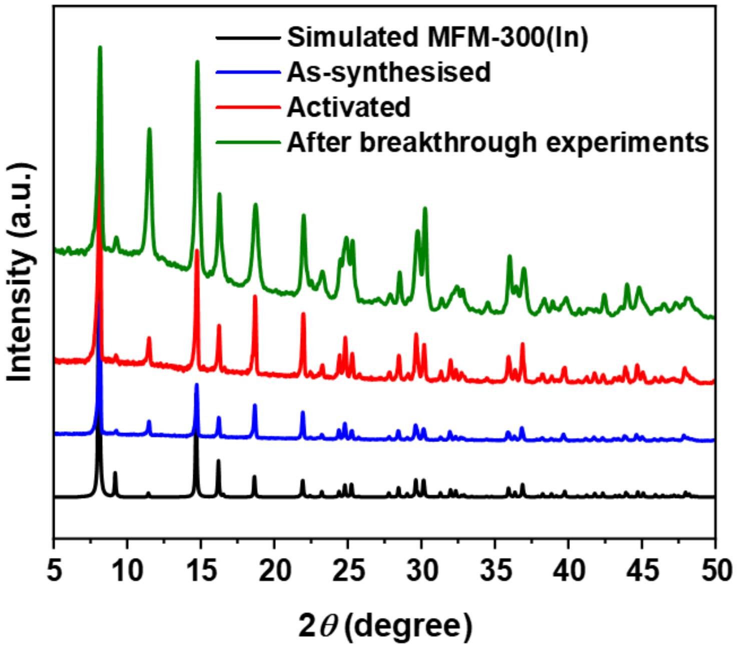

Figure S1. PXRD patterns of as-synthesised, activated MFM-300(In), and sample after breakthrough experiments.

3. Thermogravimetric Analysis

The as-synthesised, acetone exchanged and activated MFM-300(In) were heated from room temperature to 510 °C at a rate of 5 °C min^{-1} under a flow of air. The result shows that the MFM-300(In) can tolerate up to 400 °C confirming its high thermal stability.

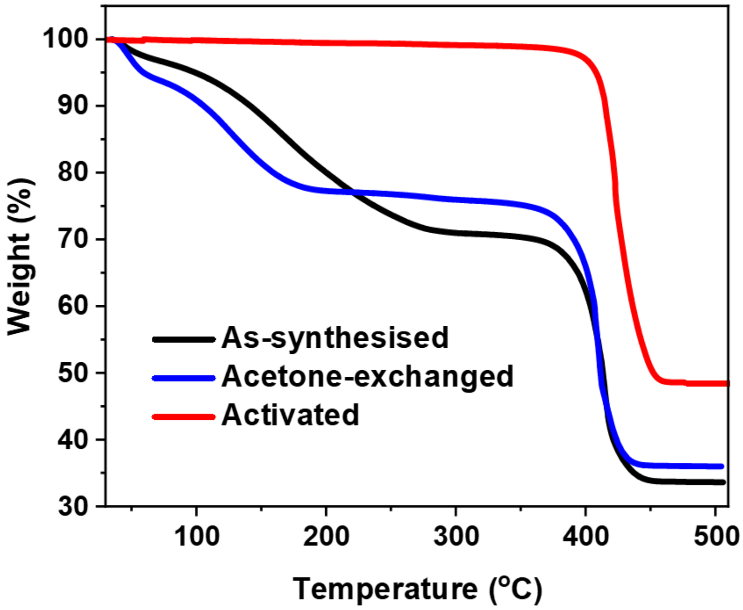

Figure S2. TGA curves for as-synthesised, acetone-exchanged and activated MFM-300(In).

4. Characterisation of Porosity

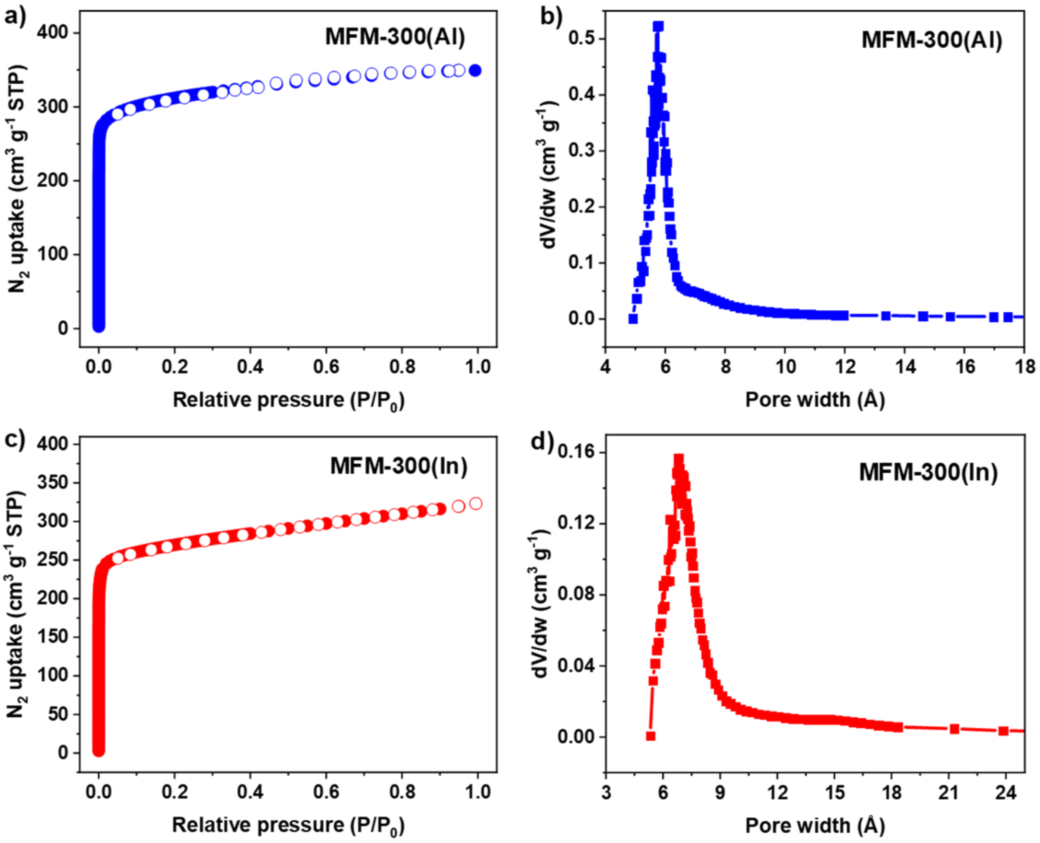

Figure S3. N_2 adsorption/desorption isotherms for a) MFM-300(Al) and c) MFM-300(In) at 77 K. Micropore size distribution plots for b) MFM-300(Al) and d) MFM-300(In).

5. Additional Gas Adsorption Isotherms

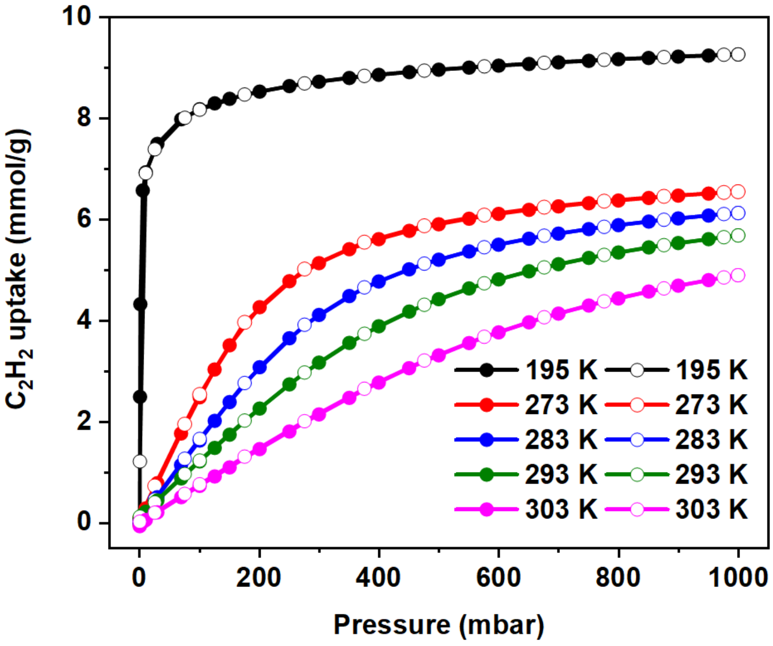

Figure S4. Adsorption/desorption isotherms for acetylene in MFM-300(In).

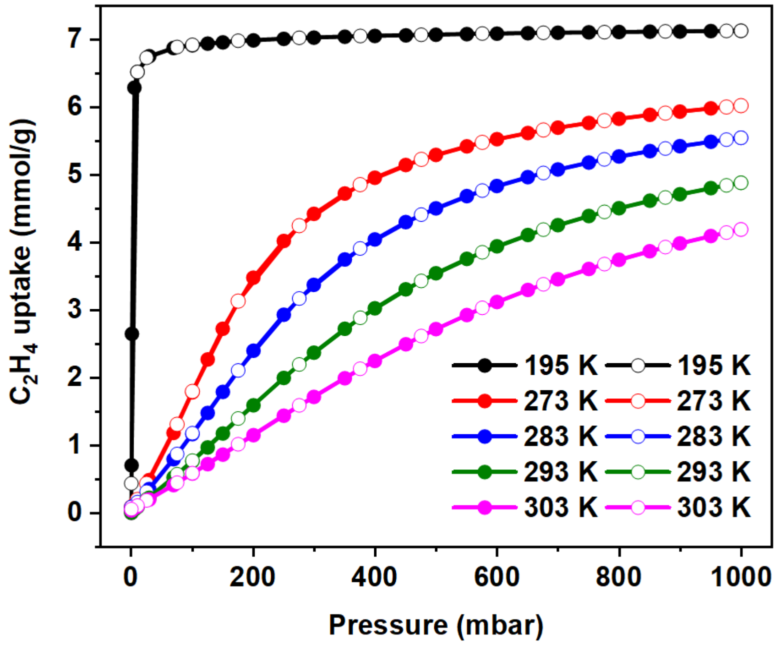

Figure S5. Adsorption/desorption isotherms for ethylene in MFM-300(In).

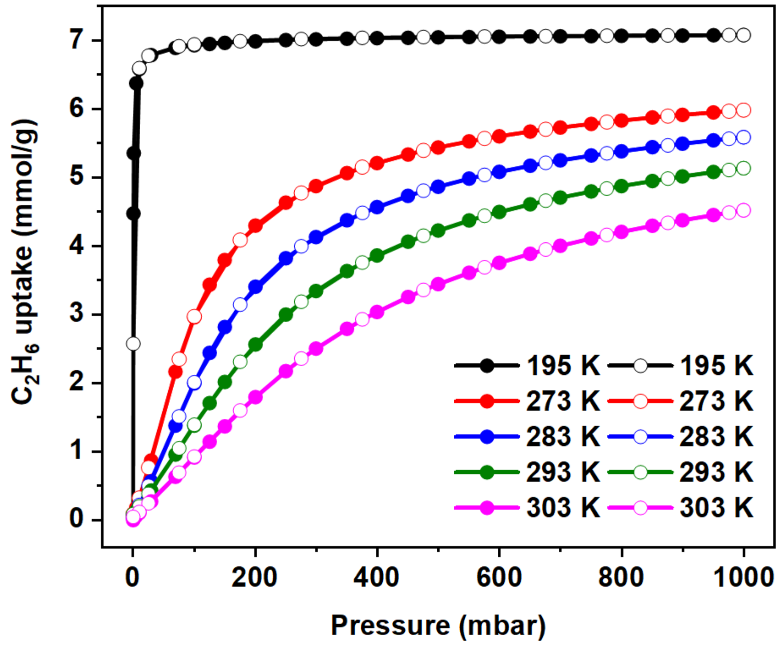

Figure S6. Adsorption/desorption isotherms for ethane in MFM-300(In).

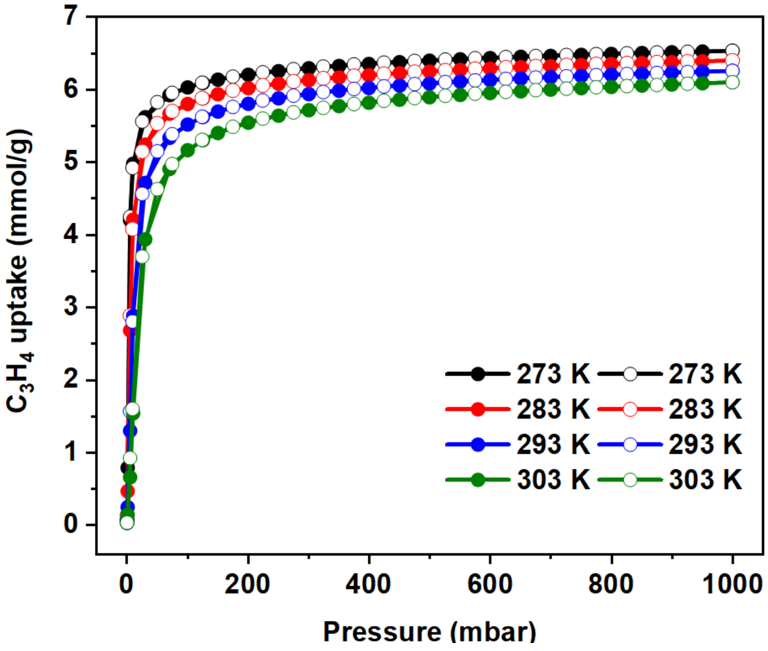

Figure S7. Adsorption/desorption isotherms for propyne in MFM-300(In).

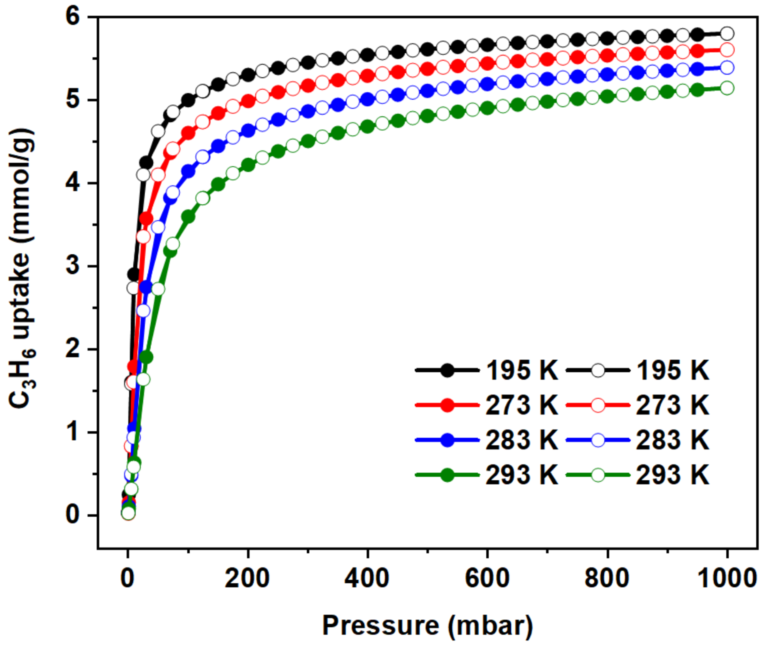

Figure S8. Adsorption/desorption isotherms for propene in MFM-300(In).

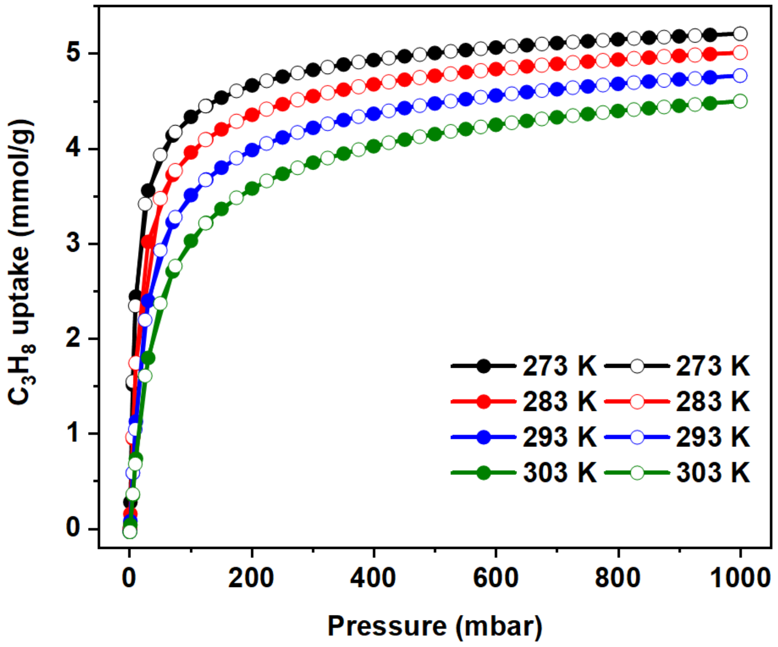

Figure S9. Adsorption/desorption isotherms for propane in MFM-300(In).

6. Comparison of C_2H_2, C_2H_4, C_2H_6, C_3H_4, C_3H_6 and C_3H_8 isotherms

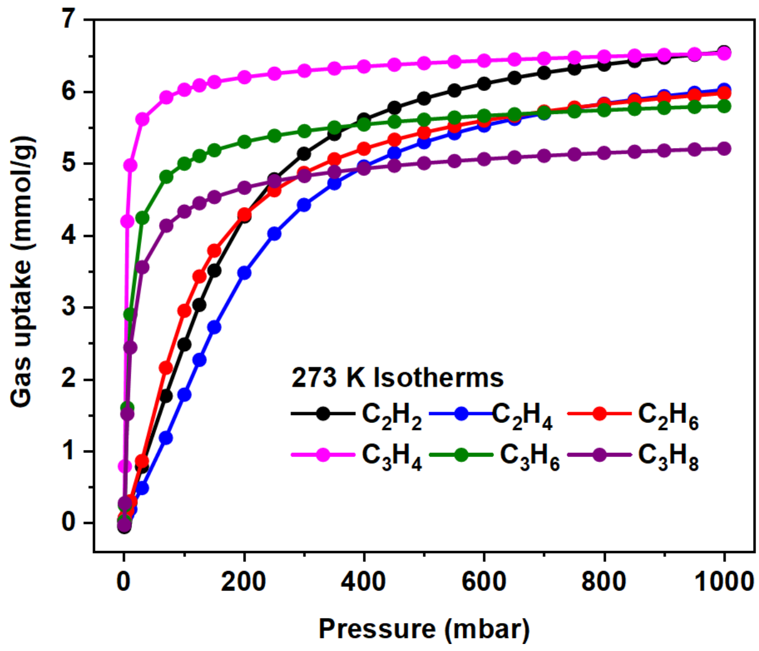

Figure S10. Adsorption isotherms at 273 K of C_2H_2, C_2H_4, C_2H_6, C_3H_4, C_3H_6 and C_3H_8 in MFM-300(In) to a pressure of 1 bar. Desorption isotherms are omitted for clarity; the nature of reversible adsorption has been demonstrated above.

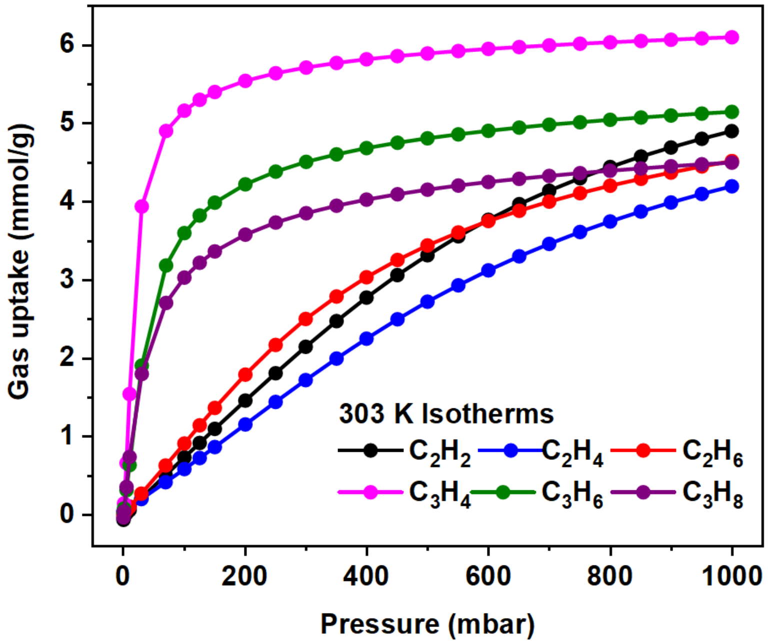

Figure S11. Adsorption isotherms at 303 K of C_2H_2, C_2H_4, C_2H_6, C_3H_4, C_3H_6 and C_3H_8 in MFM-300(In) to a pressure of 1 bar. Desorption isotherms are omitted for clarity; the nature of reversible adsorption has been demonstrated above.

7. Analysis and Derivation of the Isosteric Heats of Adsorption

To estimate the isosteric enthalpies (ΔH) for adsorption of C_2H_2, C_2H_4, C_2H_6, C_3H_4, C_3H_6 and C_3H_8 isotherms between 273–308 K were fitted to the Van t' Hoff equation;

$$\ln P = \frac{\Delta H}{RT} - \frac{\Delta S}{R} \quad (1)$$

where *p* is pressure in Pa, *T* is the temperature, and *R* is the ideal gas constant. All linear fittings show R^2 above 0.99 indicating the consistency of the isotherm data and of the fitting.

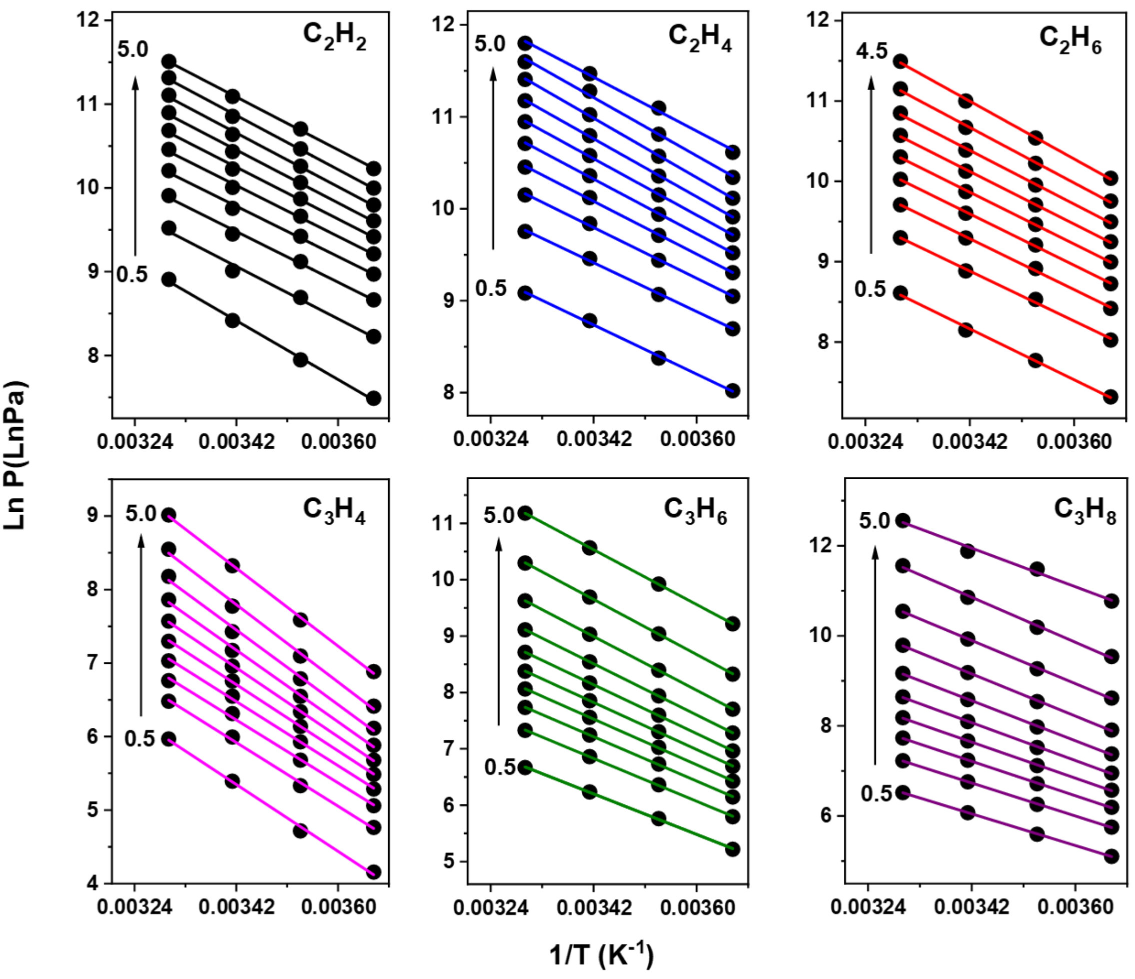

Figure S12. Linear fitting of 1/T vs LnP at intervals of 0.1 mmolg^{-1} for substrates in MFM-300(In) to determine the isosteric heat of adsorption by the Van t' Hoff method.

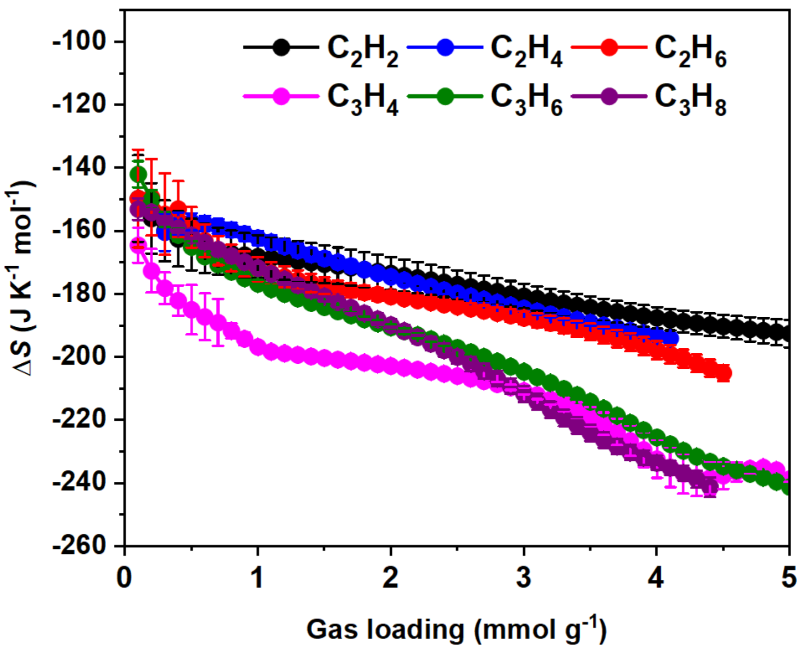

Figure S13. Entropy of adsorption for C_2 and C_3 hydrocarbons in MFM-300(In) calculated from isotherm data.

8. Calculation of IAST selectivity for gas adsorption.

To estimate the selectivity observed for each substrate isotherm data at 293 K were fitted using the dual-site Langmuir-Freundlich (DSLF) model (equation 2).

$$N^{\circ}(f) = \frac{q_1 b_1 P^{v_1}}{1 + b_1 P^{v_1}} + \frac{q_2 b_2 P^{v_2}}{1 + b_2 P^{v_2}} \quad [2]$$

where P is the pressure of the bulk gas at equilibrium with the adsorbed phase, q_i is the maximum adsorption amount, b_i is the the affinity constant and n_i is the deviation from the simple Langmuir equation. Using this fitting, the IAST selectivity can be calculated by equation 3.

$$S = \frac{x_1/y_1}{x_2/y_2} \quad [3]$$

where x_{i} is the amount of each component adsorbed and y_{i} is the mole fraction of each component at equilibrium.

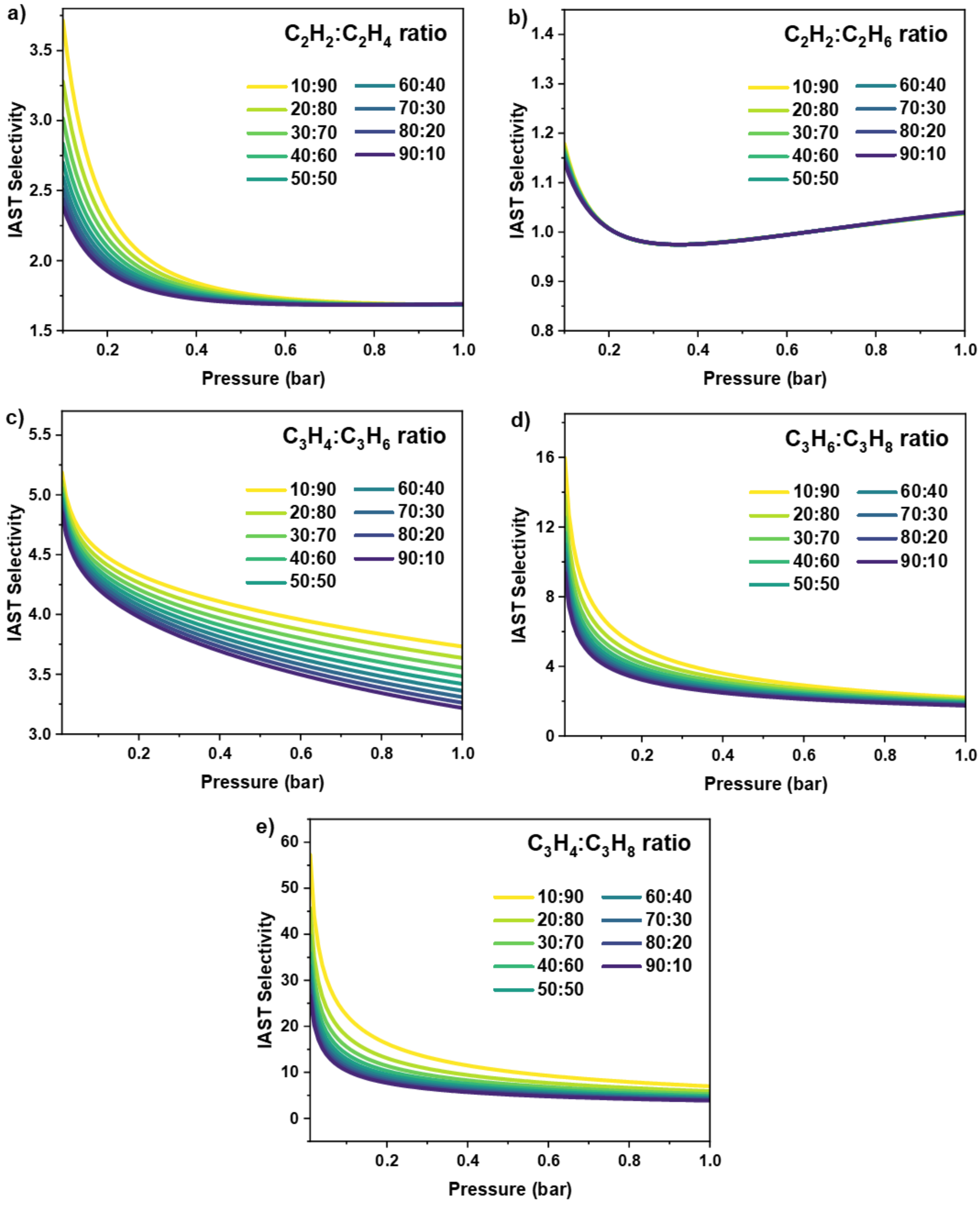

Figure S14. Selectivities as a function of pressure for C_2 and C_3 hydrocarbons in MFM-300(In) calculated by IAST from single component adsorption isotherms.

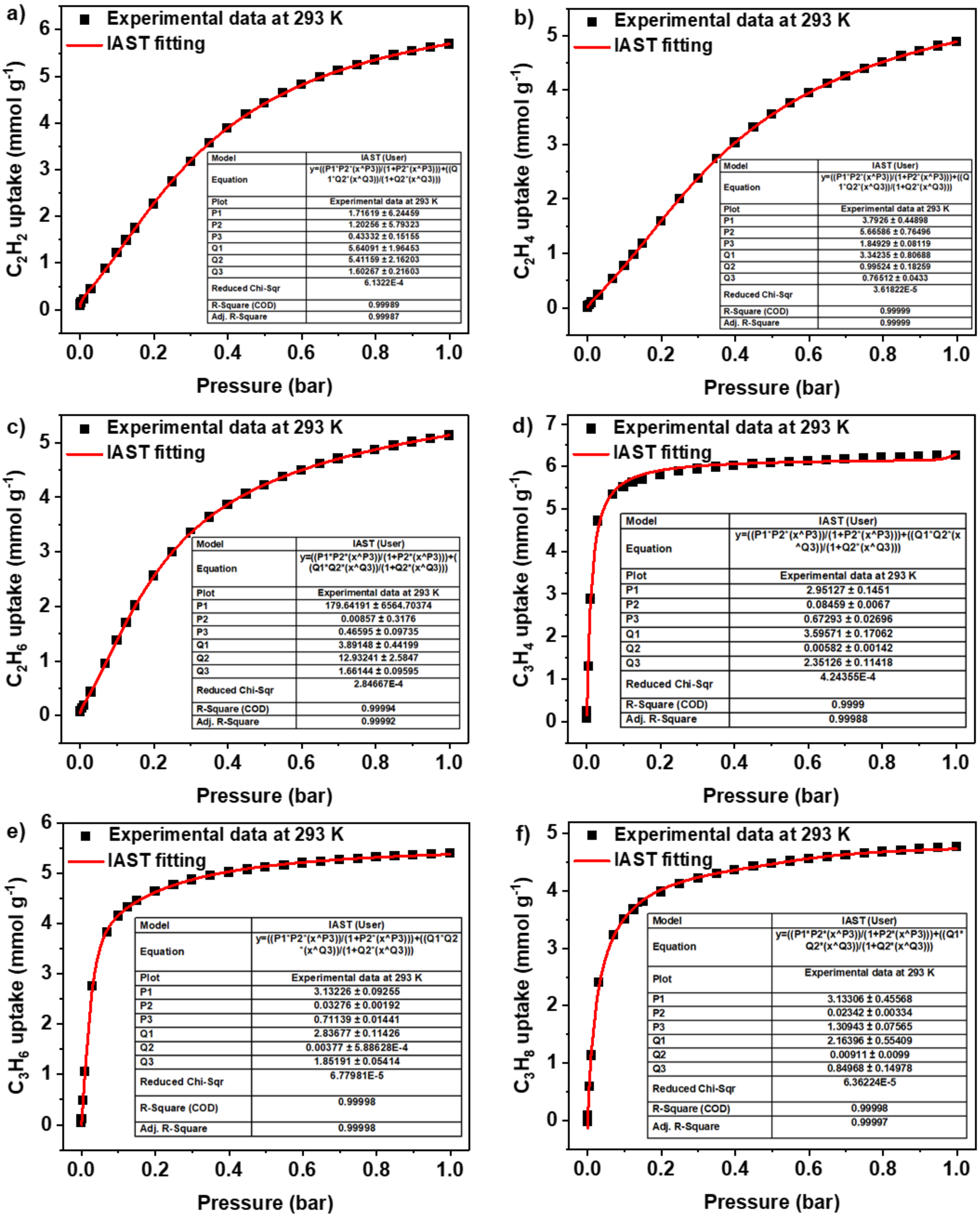

Figure S15. IAST fitting of isotherms for (a) C_2H_2, (b) C_2H_4, (c) C_2H_6, (d) C_3H_4, (e) C_3H_6 and (f) C_3H_8-loaded MFM-300(In) at 293 K and up to 1 bar.

9. Dynamic Breakthrough Experiments

Calculation of dynamic adsorption capacity and productivity

To determine the dynamic adsorption capacity, the uptake of each component (*n_m*) was calculated based on the breakthrough curves by the equation described as follows:

$$V_m = \frac{\int_0^t v_{gas\ out}\, d_t - V_{dead}}{W_{MOF}}$$

$$n_m = \frac{PV_m}{\mathrm{R}T}$$

where $v_{\mathrm{gas\ out}}$ is the flow rate of the target gas with the unit of mL min^{-1}; V_{dead} is the dead volume of the system (mL); *W* represents the mass of MFM-300(In) packed in the breakthrough bed (g); *t* is the retention time for the specific gas (min); *P* is atmospheric pressure (Kpa); R is *Avogadro* constant. *T* is the measurement temperature (K).

The productivity (q_{m}) of C_2H_4 and C_3H_6 was determined through the breakthrough amount of C_2H_4 and C_3H_6, which is calculated by integration of the breakthrough curves during a period from t_1 to t_2 during which the gas purity is greater than 99.9%:

$$q_m = \frac{\int_{t1}^{t2} v_{gas\ out}\, d_t - V_{dead}}{W_{MOF}}$$

where $v_{\mathrm{gas\ out}}$ is the flow rate of target gas with the unit of mL min^{-1}; V_{dead} is the dead volume of the system (mL); *W* represents the mass of MFM-300(In) packed in the breakthrough bed (g);

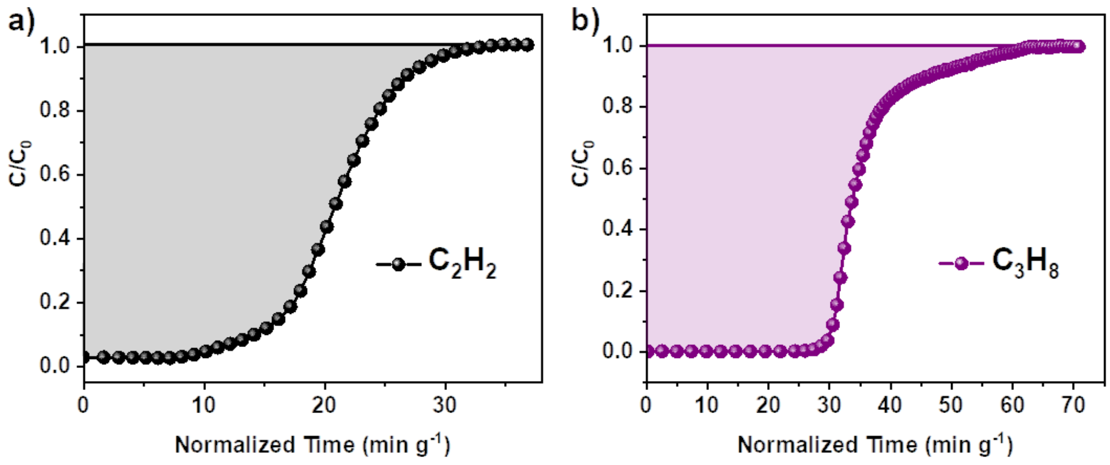

Figure S16. Breakthrough plots for single component (a) C_2H_2 and (b) C_3H_8 with an inlet gas flow rate of 2.0 mL min^{-1} diluted in He through MFM-300(In) at a total flow of 20 mL min^{-1} at 293 K.

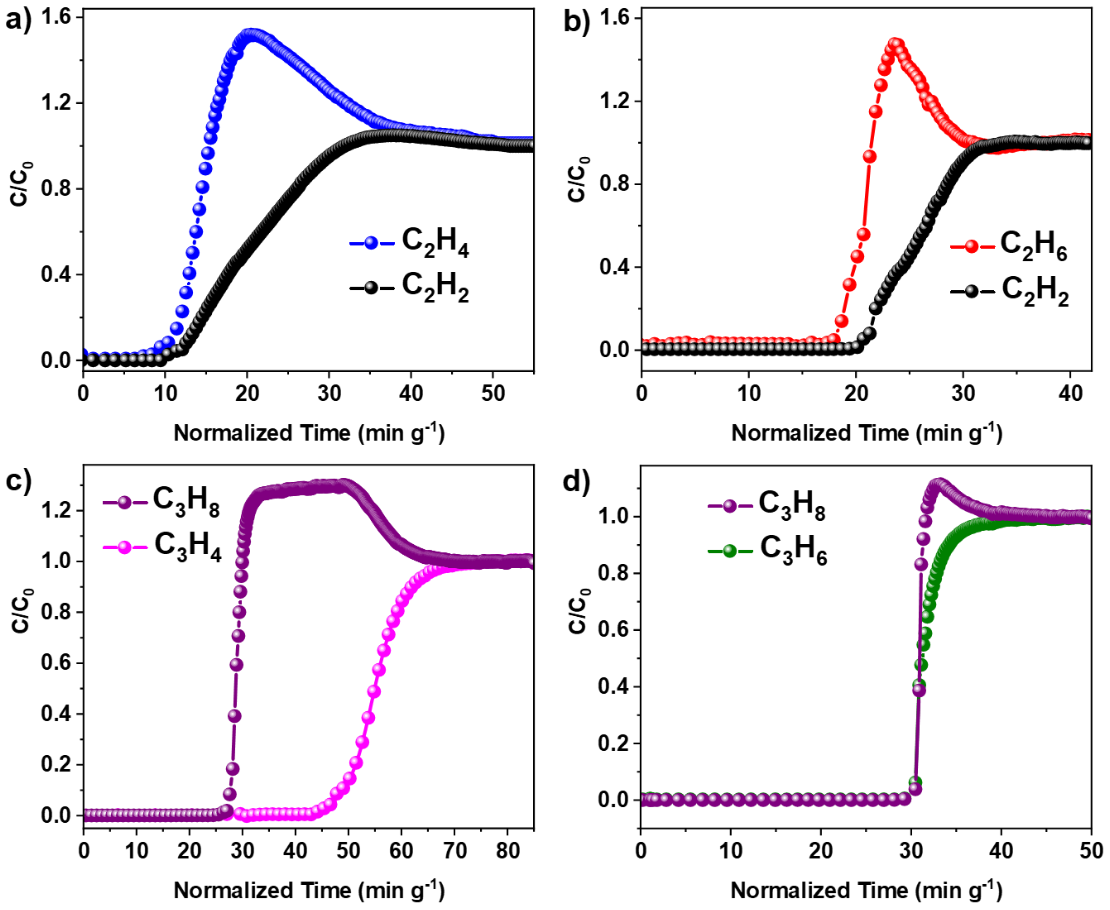

Figure S17. Dynamic breakthrough plots for equimolar mixtures of (a) C_2H_4/C_2H_2, (b) C_2H_6/C_2H_2, (c) C_3H_8/C_3H_4 and (d) C_3H_8/C_3H_6 with an inlet gas flow rate of 2.0 mL min^{-1}/2.0 mL min^{-1} diluted in He through a fixed-bed packed with MFM-300(In) at a total flow of 20 mL min^{-1} at 293 K.

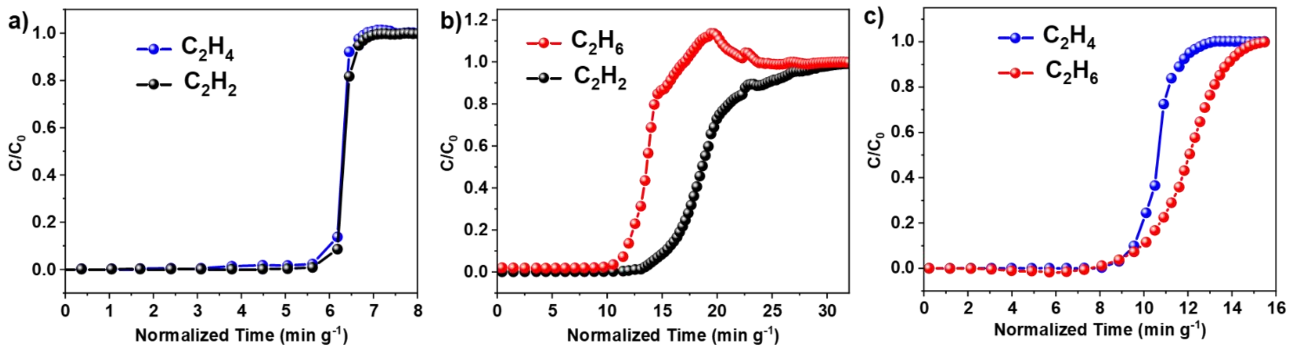

Figure S18. Dynamic breakthrough experiments for 1:99 mixtures of (a) C_2H_2/C_2H_4, (b) C_2H_2/C_2H_6, and (c) C_2H_4/C_2H_6 with an inlet gas flow rate of 0.2 mL min^{-1}/19.8 mL min^{-1} through a fixed-bed packed with MFM-300(In) at a total flow rate of 20 mL min^{-1} at 293 K.

Table S2. Dynamic adsorption of substrates on MFM-300(In) based on the breakthrough experiments.

	Amount adsorbed (mmol g^{-1})
C_2H_2	1.4
C_2H_4	1.0
C_2H_6	1.6
C_3H_4	4.4
C_3H_6	3.5
C_3H_8	3.1
C_2H_4 in equimolar C_2H_6/C_2H_4 mixture	0.7
C_2H_6 in equimolar C_2H_6/C_2H_4 mixture	1.4
C_3H_4 in equimolar C_3H_4/C_3H_6 mixture	4.6
C_3H_6 in equimolar C_3H_4/C_3H_6 mixture	3.1

Table S3. Comparsion of separation performance for state-of-the-art MOFs.

MOF	Pore size (Å)	Pore volume (cm^3 g^{-1})	BET surface area (m^2 g^{-1})	T (K)	Uptake (mmol g^{-1}) C_2H_6/C_2H_4	Selectivity C_2H_6/C_2H_4 : 50/50	Qst (KJ mol^{-1}) C_2H_6/C_2H_4	C_2H_4 Productivity (L/kg)
MFM-300(In) This work	6.8	0.43	1030	293 K	5.1/4.9	1.7	30/28	4.6 L/kg
MFM-300(Al)[9]	6.5	0.43	1370	293 K	0.85/4.28	/		/
JNU-2[10]	3.4, 4.6, 6.7 Å	0.56	1219	298 K	4.19/3.68	1.6	/	21.2 L/kg
TJT-100[11]	8.7 × 11.6	0.39	890	298 K	~3.66/3.4	1.2	29/25	/
IRMOF-8[12]	17.5	0.69	1360	298 K	2.16/1.25	1.8	52.5/50	2.5 L/kg
PCN-250[13]	5.9, 6.8, 9.3	0.56	1470	298 K	5.21/4.22	1.9	23/21	10 L/kg
MUF-15[14]	8.5 × 3.5, 7 × 3.8, 3.2 × 1.2	0.51	1130	293 K	4.69/4.15	1.96	28.2/29.2	14 L/kg
$Cu(Qc)_2$[15]	3.3	0.11	240	298 K	1.85/0.78	3.4	29/25.4	4.3 L/kg
$Ni(bdc)(ted)_{0.5}$[16]	7.94	0.79	1701	298 K	5.0/3.4	2	21.5/18.2	/
PCN-245[17]	10	0.71	1743	298 K	3.27/2.39	1.9	20.5/23.0	5.8 L/kg
$Fe_2(O_2)dobdc$[18]	/	/	1073	298 K	3.45/2.68	4.4		19.3 L/kg
ZIF-4[19]	/	0.38	300	293 K	2.3/2.2	1.7	/	
ZIF-8[20]	3.4	0.73	1844	293 K	2.54/1.5	1.8	17.2/16.1	/

11. Neutron Powder Diffraction

Neutron powder diffraction experiments were undertaken at the WISH diffractometer at the ISIS Facility. MFM-300(In) was loaded into a 6 mm diameter vanadium sample can and outgassed at 1×10^{-7} mbar and 100 °C for 1 day. The sample was loaded into a liquid helium cryostat and cooled to 7 K for data collection. C_2H_2, C_2H_4, C_2H_6, C_3H_4, C_3H_6 and C_3H_8 gas were introduced by warming the samples to 298 K and the gas dosed volumetrically from a calibrated volume. The gas-loaded sample was then cooled to 7 K over a period of 2 h to ensure good mobility of adsorbed species within the crystalline structure of MFM-300(In) and for a further 30 mins to ensure thermal equilibrium. Rietveld structural refinements were carried out on the NPD data using the TOPAS software package.[21]

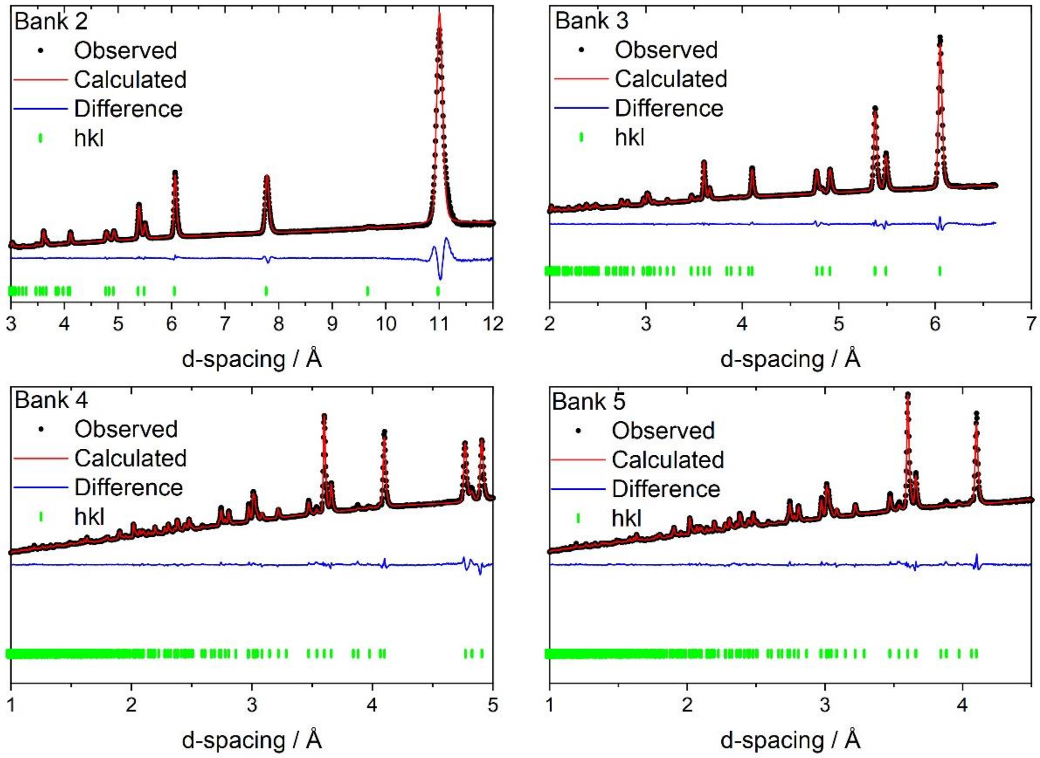

Figure S19. Rietveld fit profiles of the NPD data of MFM-300(In)·1.32(C_2D_2).

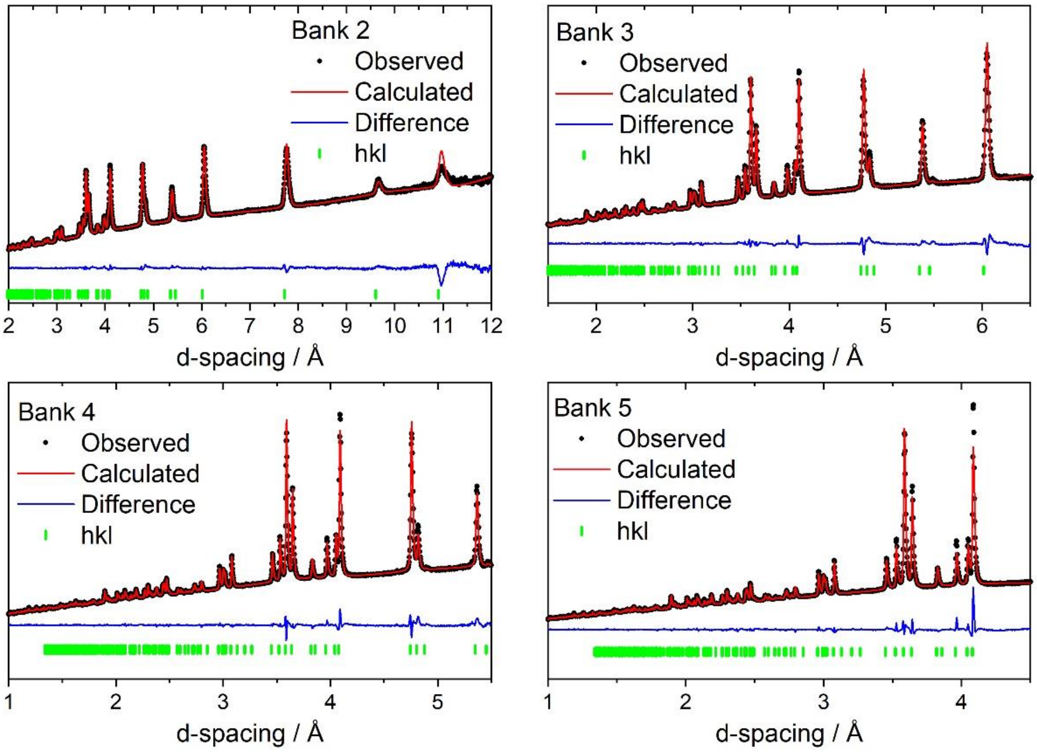

Figure S20. Rietveld fit profiles of the NPD data of MFM-300(In)·1.66(C_2D_4)

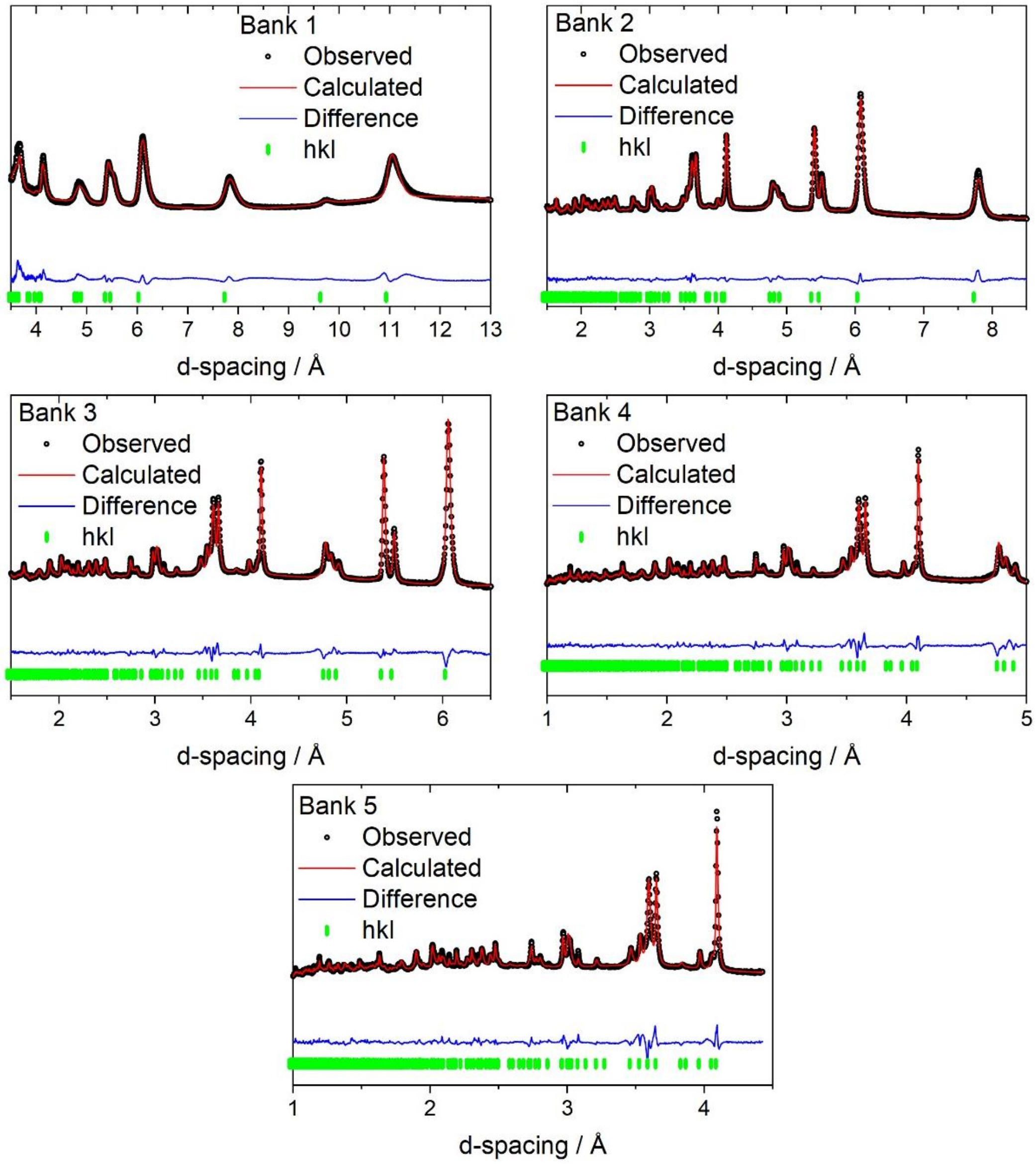

Figure S21. Rietveld fit profiles of the NPD data of MFM-300(In)·0.72(C_2D_6).

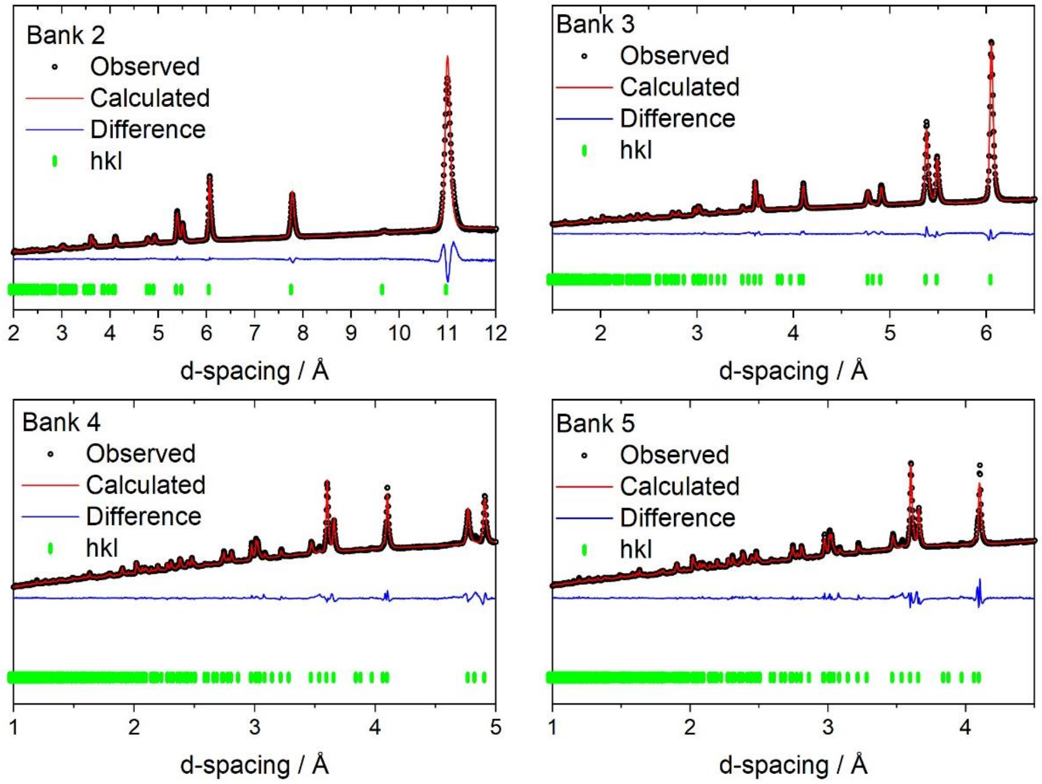

Figure S22. Rietveld fit profiles of the NPD data of MFM-300(In)·0.2(C_3D_4).

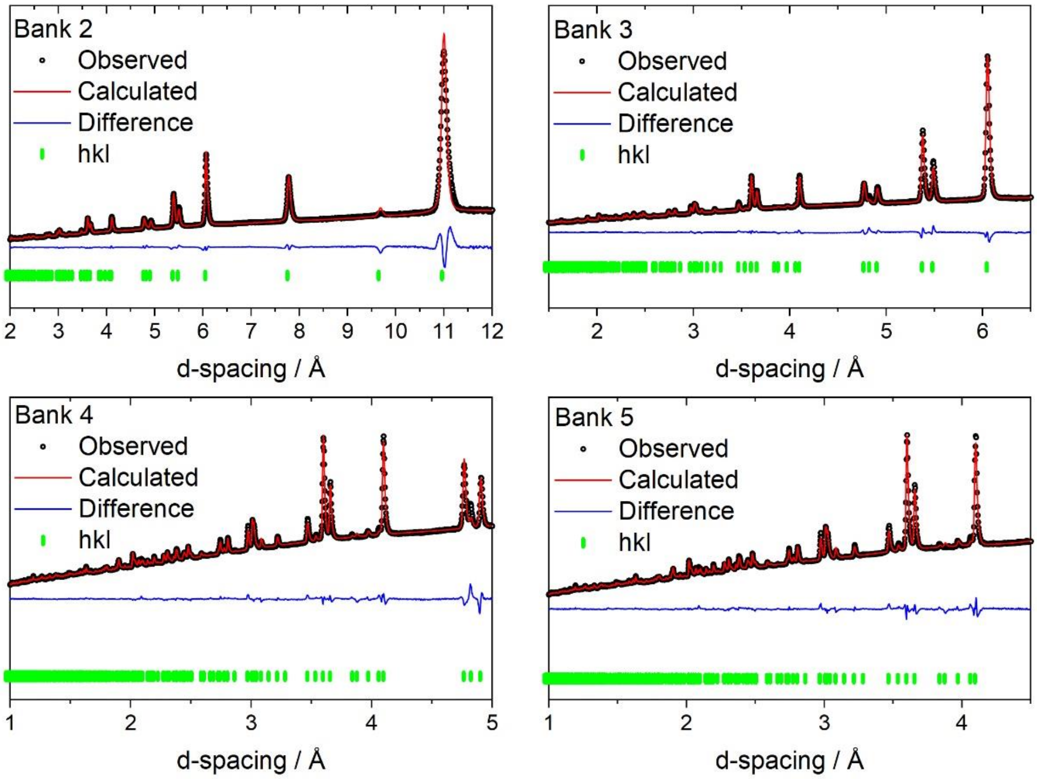

Figure S23. Rietveld fit profiles of the NPD data of MFM-300(In)·0.48(C_3D_6).

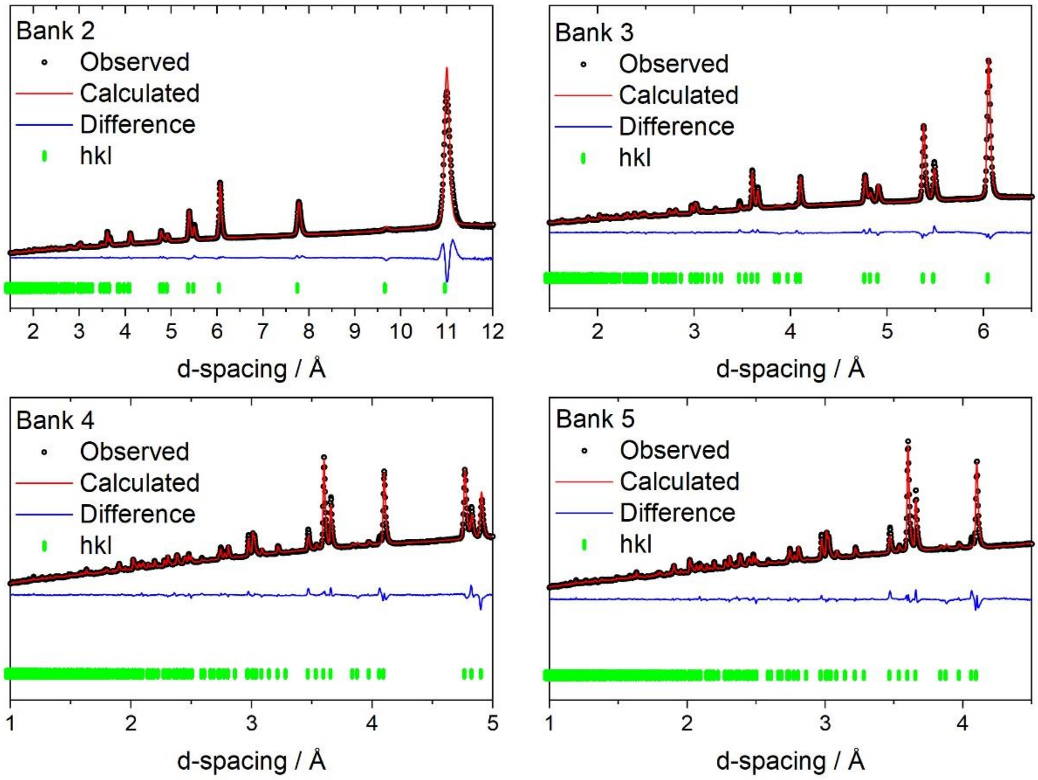

Figure S24. Rietveld fit profiles of the NPD data of MFM-300(In)·0.46(C_3D_8).

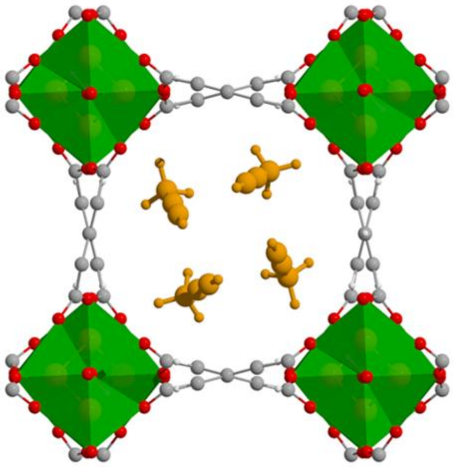

Figure S25. NPD structure of MFM-300(In)·0.2(C_3D_4).

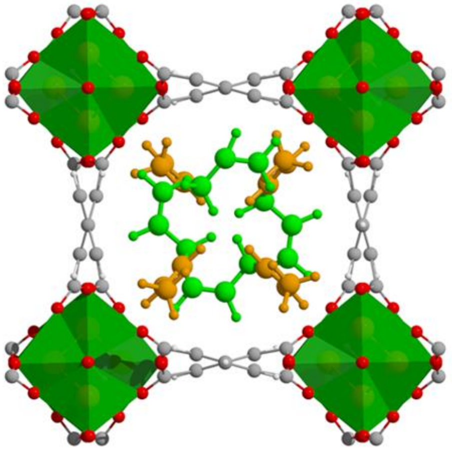

Figure S26. NPD structure of MFM-300(In)·0.48(C_3D_6).

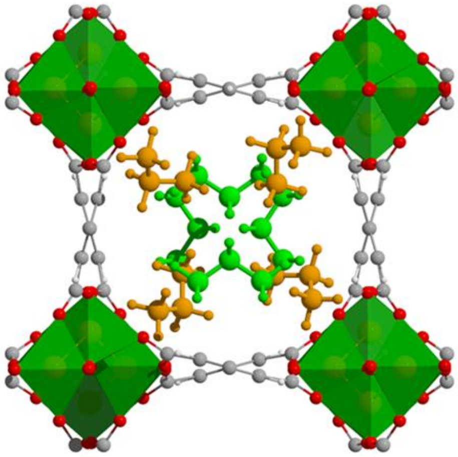

Figure S27. NPD structure of MFM-300(In)·0.46(C_3D_8).

Table S4. Host–Guest Interactions in MFM-300(In)·1.32(C_2D_2).

MFM-300(In)·1.32(C_2D_2)	Interactions	Distances (Å)	Colour
Site I	H (HO-In)···C≡C (site I)	2.52(1)	Violet
	H (site I)···C≡C (site II)	3.73(1)	Bright green
	C≡C (site I)···phenyl groups	3.83(1)	Orange
		4.04(1)	
Site II	C≡C (site II)···H (site I)	3.73(2)	Bright green

Table S5. Host–Guest Interactions in MFM-300(In)·1.66(C_2H_4).

MFM-300(In)·1.66(C_2H_4)	Interactions	Distances (Å)	Colour
Site I	H (HO-In)···C=C (site I)	3.85(1)	Violet
	H (site I)···C=C (site II)	3.91(1)	Bright green
		4.01(1)	
	H (site I)···phenyl groups	2.92(1)	Orange
		3.03(1)	
		3.73(2)	
		4.40(1)	
Site II	C=C (site II)···H (site I)	3.91(1)	Bright green
		4.01(1)	
	H (site II)···phenyl groups	4.27(1)	Pink

Table S6. Host–Guest Interactions in MFM-300(In)·0.72(C_2H_6).

MFM-300(In)·0.72(C_2H_6)	Interactions	Distances (Å)	Colour
Site I	H (HO-In)···C (site I)	3.22(2)	Violet
	H (site I)···C (site II)	2.99(4)	Bright green
	H (site I)···phenyl groups	2.65(2)	Orange
		3.30(2)	
		3.68(1)	
		4.18(2)	
Site II	C (site II)··· H (site I)	2.99(4)	Bright green

Table S7. Host–Guest Interactions in MFM-300(In)·0.2(C_3H_4).

MFM-300(In)·0.2(C_3H_4)	Interactions	Distances (Å)	Colour
Site I	H (HO-In)···C_3D_4	3.26(6)	Violet
	H (site I)···phenyl groups	3.18(6)	Orange
	C≡C (site I)··· phenyl groups	3.56(1)	Green

Table S8. Host–Guest Interactions in MFM-300(In)·0.48(C_3H_6).

MFM-300(In)·0.48(C_3H_6)	Interactions	Distances (Å)	Colour
Site I	H (HO-In)···C=C (site I)	3.37(1)	Violet
	H (site I)···phenyl groups	3.03(2)	Orange
		4.17(2)	
	C=C (site I) ··· phenyl groups	3.89(1)	Blue
Site II	C=C (site II)··· H (site I)	1.91(2)	Bright green
	H (siteII)···phenyl groups	4.07(1)	Orange

Table S9. Host–Guest Interactions in MFM-300(In)·0.46(C_3H_8).

MFM-300(In)·0.46(C_3H_8)	Interactions	Distances (Å)	Colour
Site I	H (HO-In)···C (site I)	2.72(2) Å	Violet
	H (site I)···C (site II)	2.92(2)	Bright green
	C (site I)···H (site II)	3.19(2)	
	H (siteI)···phenyl groups	3.17(2)	Orange
		4.87(2)	
		4.02 (2)	
		3.37(2)	
Site II	C (site II)···H (site I)	2.92(2)	Bright green
	C (site I)···H (site II)	3.19(2)	
	H (site II)··· phenyl groups	3.35(1)	Pink
		3.02(1)	

12. Inelastic Neutron Scattering Measurement

Inelastic neutron scattering (INS) experiments were undertaken using the TOSCA spectrometer at the ISIS Facility. MFM-300(In) was loaded into an 11 mm diameter vanadium sample can and outgassed at 1×10^{-7} mbar and 100 °C for 1 day. The sample was loaded into a helium closed cycle refrigerator (CCR) cryostat and cooled to 11 K for data collection. C_2H_2, C_2H_4 and C_2H_6 gas were introduced by warming the sample to 298 K and the gas was dosed volumetrically from a calibrated volume. The gas-loaded sample was then cooled to 7 K over a period of 2 h to ensure good mobility of adsorbed species within the crystalline structure of MFM-300(In). The sample was kept at 7 K for an additional 30 mins before data collection to ensure the thermal equilibrium.

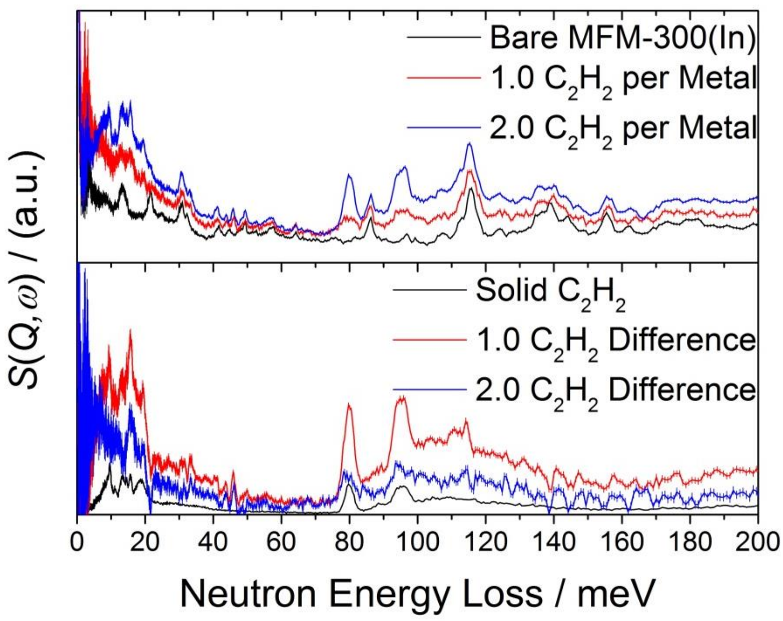

Figure S28. Comparison of bare and C_2H_2 loaded MFM-300(In).

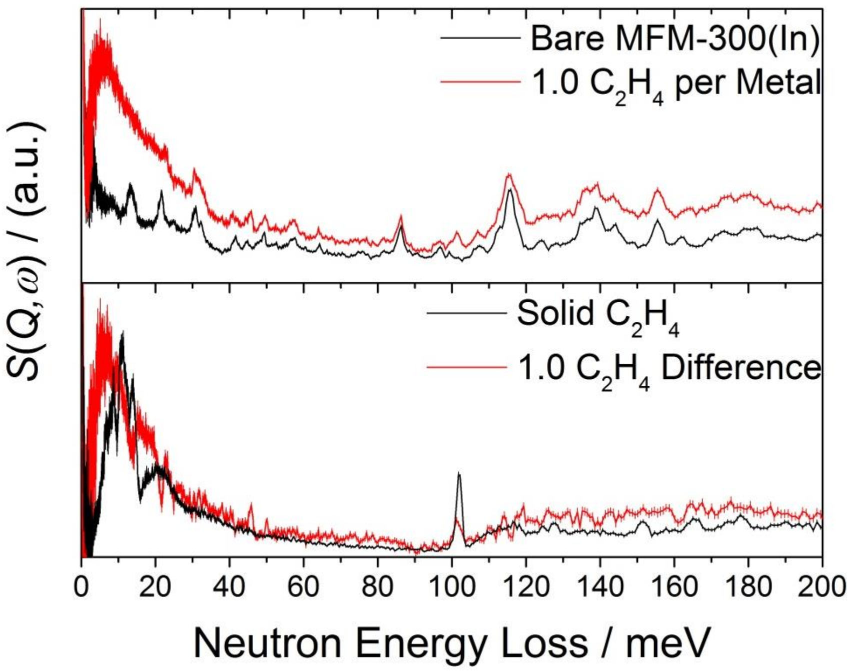

Figure S29. Comparison of bare and C_2H_4 loaded MFM-300(In).

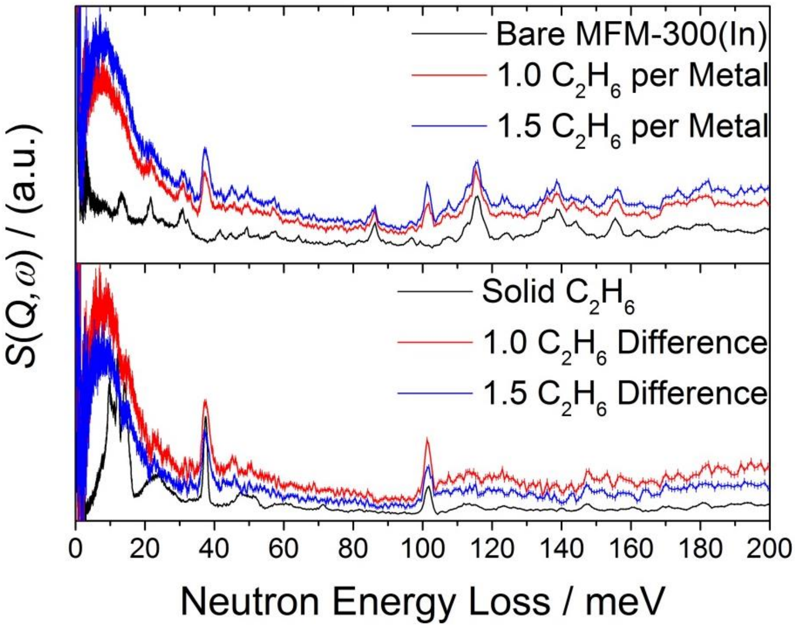

Figure S30. Comparison of bare and C_2H_6 loaded MFM-300(In).

13. References

1. Savage, M.; Cheng, Y.; Easun, T. L.; Eyley, J. E.; Argent, S. P.; Warren, M. R.; Lewis, W.; Murray, C.; Tang, C. C.; Frogley, M. D.; Cinque, G.; Sun, J.; Rudic, S.; Murden, R. T.; Benham, M. J.; Fitch, A. N.; Blake, A. J.; Ramirez-Cuesta, A. J.; Yang, S.; Schröder, M., Selective Adsorption of Sulfur Dioxide in a Robust Metal-Organic Framework Material. *Adv. Mater.* **2016,** *28*, 8705–8711.

2. Li, J. R.; Kuppler, R. J.; Zhou, H. C., Selective Gas Adsorption and Separation in Metal-Organic Frameworks. *Chem. Soc. Rev.* **2009,** *38*, 1477–1504.

3. Geng, S.; Lin, E.; Li, X.; Liu, W.; Wang, T.; Wang, Z.; Sensharma, D.; Darwish, S.; Andaloussi, Y. H.; Pham, T.; Cheng, P.; Zaworotko, M. J.; Chen, Y.; Zhang, Z., Scalable Room-Temperature Synthesis of Highly Robust Ethane-Selective Metal-Organic Frameworks for Efficient Ethylene Purification. *J. Am. Chem. Soc.* **2021,** *143*, 8654–8660.

4. Wang, S.-M.; Wang, F.; Dong, Y.-L.; Shivanna, M.; Dong, Q.; Mu, X.-T.; Duan, J.; Yang, Q.; Zaworotko, M. J.; Yang, Q.-Y., Reversed C_2H_6/C_2H_4 Separation in Interpenetrated Diamondoid Coordination Networks with Enhanced Host–Guest Interaction. *Sep. Purif. Technol.* **2021,** *276*, 119385.

5. Liu, P.; Wang, Y.; Chen, Y.; Yang, J.; Wang, X.; Li, L.; Li, J., Construction of Saturated Coordination Titanium-Based Metal–Organic Framework for One-Step $C_2H_2/C_2H_6/C_2H_4$ Separation. *Sep. Purif. Technol.* **2021,** *276*, 119284.

6. Gao, J.; Qian, X.; Lin, R. B.; Krishna, R.; Wu, H.; Zhou, W.; Chen, B., Mixed Metal-Organic Framework with Multiple Binding Sites for Efficient C_2H_2/CO_2 Separation. *Angew. Chem. Int. Edit.* **2020,** *59*, 4396–4400.

7. Ding, Q.; Zhang, Z.; Yu, C.; Zhang, P.; Wang, J.; Kong, L.; Cui, X.; He, C. H.; Deng, S.; Xing, H., Separation of Propylene and Propane with a Microporous Metal-Organic Framework via Equilibrium–Kinetic Synergetic Effect. *AIChE J.* **2020,** *67*, 17094.

8. Kim, S.-J.; Lee, P. S.; Chang, J.-S.; Nam, S.-E.; Park, Y.-I., Preparation of Carbon Molecular Sieve Membranes on Low-Cost Alumina Hollow Fibers for Use in C_3H_6/C_3H_8 Separation. *Sep. Purif. Technol.* **2018,** *194*, 443–450.

9. Yang, S.; Ramirez-Cuesta, A. J.; Newby, R.; Garcia-Sakai, V.; Manuel, P.; Callear, S. K.; Campbell, S. I.; Tang, C. C.; Schröder, M., Supramolecular Binding and Separation of Hydrocarbons within a Functionalized Porous Metal-Organic Framework. *Nat. Chem.* **2015,** *7*, 121–129.

10. Zeng, H.; Xie, X. J.; Xie, M.; Huang, Y. L.; Luo, D.; Wang, T.; Zhao, Y.; Lu, W.; Li, D., Cage-Interconnected Metal-Organic Framework with Tailored Apertures for Efficient C_2H_6/C_2H_4 Separation under Humid Conditions. *J. Am. Chem. Soc.* **2019,** *141*, 20390–20396.

11. Hao, H. G.; Zhao, Y. F.; Chen, D. M.; Yu, J. M.; Tan, K.; Ma, S.; Chabal, Y.; Zhang, Z. M.; Dou, J. M.; Xiao, Z. H.; Day, G.; Zhou, H. C.; Lu, T. B., Simultaneous Trapping of C_2H_2 and C_2H_6 from a Ternary Mixture of $C_2H_2/C_2H_4/C_2H_6$ in a Robust Metal–Organic Framework for the Purification of C_2H_4. *Angew. Chem. Int. Edit.* **2018,** *130*, 16299–16303.

12. Pires, J.; Pinto, M. L.; Saini, V. K., Ethane Selective IRMOF-8 and its Significance in Ethane-Ethylene Separation by Adsorption. *ACS Appl. Mater. Interfaces* **2014,** *6*, 12093–12099.

13. Chen, Y.; Qiao, Z.; Wu, H.; Lv, D.; Shi, R.; Xia, Q.; Zhou, J.; Li, Z., An Ethane-Trapping MOF PCN-250 for Highly Selective Adsorption of Ethane over Ethylene. *Chem. Eng. J.* **2018,** *175*, 110–117.

14. Qazvini, O. T.; Babarao, R.; Shi, Z. L.; Zhang, Y. B.; Telfer, S. G., A Robust Ethane-Trapping Metal-Organic Framework with a High Capacity for Ethylene Purification. *J. Am. Chem. Soc.* **2019,** *141*, 5014–5020.

15. Lin, R. B.; Wu, H.; Li, L.; Tang, X. L.; Li, Z.; Gao, J.; Cui, H.; Zhou, W.; Chen, B., Boosting Ethane/Ethylene Separation within Isoreticular Ultramicroporous Metal-Organic Frameworks. *J. Am. Chem. Soc.* **2018,** *140*, 12940–12946.

16. Liang, W.; Xu, F.; Zhou, X.; Xiao, J.; Xia, Q.; Li, Y.; Li, Z., Ethane Selective Adsorbent $Ni(Bdc)(Ted)_{0.5}$ with High Uptake and Its Significance in Adsorption Separation of Ethane and Ethylene. *Chem. Eng. J.* **2016,** *148*, 275–281.

17. Lv, D.; Shi, R.; Chen, Y.; Wu, Y.; Wu, H.; Xi, H.; Xia, Q.; Li, Z., Selective Adsorption of Ethane over Ethylene in PCN-245: Impacts of Interpenetrated Adsorbent. *ACS Appl. Mater. Interfaces* **2018,** *10*, 8366–8373.

18. Li, L.; Lin, R. B.; Krishna, R.; Li, H.; Xiang, S.; Wu, H.; Li, J.; Zhou, W.; Chen, B., Ethane/Ethylene Separation in a Metal-Organic Framework with Iron-peroxo Sites. *Science* **2018,** *362*, 443–446.

19. Hartmann, M.; Bohme, U.; Hovestadt, M.; Paula, C., Adsorptive Separation of Olefin/Paraffin Mixtures with ZIF-4. *Langmuir* **2015,** *31*, 12382–12389.

20. Bohme, U.; Barth, B.; Paula, C.; Kuhnt, A.; Schwieger, W.; Mundstock, A.; Caro, J.; Hartmann, M., Ethene/Ethane and Propene/Propane Separation via The Olefin and Paraffin Selective Metal-Organic Framework Adsorbents CPO-27 And ZIF-8. *Langmuir.* **2013,** *29*, 8592–600.

21. Coelho, A. A., TOPAS And TOPAS-Academic: An Optimization Program Integrating Computer Algebra and Crystallographic Objects Written in C^{++}. *J. Appl. Cryst.* **2018,** *51*, 210–218.
